# Baicalin enhances the chemotherapy sensitivity of oxaliplatin-resistant gastric cancer cells by activating p53-mediated ferroptosis

**DOI:** 10.1038/s41598-024-60920-y

**Published:** 2024-05-10

**Authors:** Lihua Shao, Li Zhu, Rong Su, Chunting Yang, Xiaqing Gao, Yan Xu, Hongwei Wang, Chenglong Guo, Hailong Li

**Affiliations:** 1 Departments of Geriatrics, Affiliated Hospital of Gansu University of Chinese Medicine, Lanzhou, 730000 Gansu Province People’s Republic of China; 2 Department of Internal Medicine, First School of Clinical Medicine, Gansu University of Chinese Medicine, 35 East Dingxi Road, Chengguan, Lanzhou, 730000 Gansu People’s Republic of China; 3 Key Laboratory of Gansu Provincial Prescription Mining and Innovative Translational Laboratory, Gansu University of Chinese Medicine, Lanzhou, 730000 Gansu Province People’s Republic of China; 4 Emergency Department, Minda Hospital of Hubei Minzu University, Enshi, 445000 Hubei Province People’s Republic of China; 5 Department of Osteoporosis, Affiliated Hospital of Gansu University of Chinese Medicine, 730000 Lanzhou, People’s Republic of China

**Keywords:** Gastric cancer, Multidrug resistance, Baicalin, Ferroptosis, p53 suppressor gene, Cancer, Drug discovery

## Abstract

Gastric cancer is one of the most common malignant tumors, and chemotherapy is the main treatment for advanced gastric cancer. However, chemotherapy resistance leads to treatment failure and poor prognosis in patients with gastric cancer. Multidrug resistance (MDR) is a major challenge that needs to be overcome in chemotherapy. According to recent research, ferroptosis activation is crucial for tumor therapeutic strategies. In this work, we explored the solution to chemoresistance in gastric cancer by investigating the effects of the Chinese medicine monomer baicalin on ferroptosis. Baicalin with different concentrations was used to treat the parent HGC27 and drug-resistant HGC27/L cells of gastric cancer. Cell viability was measured by CCK8, and synergistic effects of baicalin combined with oxaliplatin were evaluated using Synergy Finder software. The effects of baicalin on organelles and cell morphology were investigated using projective electron microscopy. Iron concentration, MDA production and GSH inhibition rate were measured by colorimetry. ROS accumulation was detected by flow cytometry. The ferroptosis-related genes (IREB2, TfR, GPX4, FTH1), P53, and SLC7A11 were analysed by Western blot, and the expression differences of the above proteins between pretreatment and pretreatment of different concentrations of baicalin, were assayed in both parental HGC27 cells and Oxaliplatin-resistant HGC27/L cells. Mechanically, Baicalin disrupted iron homeostasis and inhibits antioxidant defense, resulting in iron accumulation, lipid peroxide aggregation, and specifically targeted and activated ferroptosis by upregulating the expression of tumor suppressor gene p53, thereby activating the SLC7A11/GPX4/ROS pathway mediated by it. Baicalin activates ferroptosis through multiple pathways and targets, thereby inhibiting the viability of oxaliplatin-resistant gastric cancer HGC27/L cells and enhancing the sensitivity to oxaliplatin chemotherapy.

## Introduction

Gastric cancer is the fourth most common malignancy in the world, and chemotherapy is the primary treatment for advanced gastric cancer. Advanced gastric cancer patients can notably benefit from chemotherapy including platinum drugs, 5-fluorouracil, doxorubicin, vincristine, and paclitaxel as well as targeted therapy drugs^1^. However, primary or acquired drug resistance ultimately leads to treatment failure and adverse outcomes in patients with gastric cancer^2^. Tumor drug resistance is one of the main causes of tumor treatment failure^3^, which greatly limited the choice and application of anti-cancer drugs, and repeatedly dashes the hope of cancer patients to be cured time and again. Highly resistant cells are usually a complex of multiple resistance mechanisms. A variety of treatment strategies have been shown to be effective in overcoming drug resistance, including multi-drug combination therapy, Initiating treatment at an early stage with a low tumor burden, or the application of new drugs to overcome drug resistance. Of course, the development of novel drugs specifically targeting tumor resistance mechanisms and the combination of multiple targeted drugs remain the main strategies to address the problem of drug resistance. Research on cancer drug resistance is crucial, and it is urgent to explore the mechanism of cancer drug resistance and new ways to overcome it.

Traditional Chinese medicine (TCM) has the advantages of multi-pathway, multi-target and safety in the treatment of diseases ^4^. Baicalin is an effective component of the Chinese herbs Scutellaria baicalensis ^5^. Previous studies have found that baicalin has significant inhibition action on various tumors, such as bladder cancer ^6^, stomach cancer^7^, lung cancer^8^, ovarian cancer^9^, liver cancer^10^ and breast cancer^11^. Its anti-tumor mechanisms are varied, including inducing apoptosis, regulating different signaling pathways, regulating autophagy and inhibiting tumor cell invasion and migration^5,12,13^. Whether baicalin demonstrates chemotherapy sensitization effect on oxaliplatin resistant gastric cancer cells or not and the underlying mechanisms are still unknown.

Ferroptosis is a new way of programmed cell death that is dependent on iron metabolism, that is, under the action of ferrobivalent or lipoxylate, it catalyzes the highly expressed unsaturated fatty acids on the cell membrane, and causes the accumulation of reactive oxygen species (ROS) leading to lipid peroxidation, thus inducing cell death^14,15^. Extensive studies suggest that ferroptosis plays a pivotal role in tumor suppression, thus providing new opportunities for cancer therapy ^16^. Alterations in signaling pathways associated with ferroptosis may reverse chemotherapy resistance. Previous studies have shown that inhibition of ferroptosis could promote chemotherapy resistance of gastric cancer^17^, and promoting ferroptosis can restore the sensitivity of gastric cancer to chemotherapies ^18^.

The aim of this study is to investigate whether baicalin can improve the chemotherapy sensitivity of oxaliplatin resistant human gastric cancer cells HCG27/L to oxaliplatin, whether Baicalin can induce ferroptosis, and to explore its potential mechanism.

## Materials and methods

### Cell lines and reagents

Human parental gastric cancer HGC-27 cell line and drug-resistant HGC27/L cell line (purchased from Shanghai Meishen Biological Company, China) were cultured in RMI-1640 and supplemented with 20% fetal bovine serum (TransGen Biotech,Beijing,china). The Drug-resistant HGC27/L cell lines were regularly stimulated with oxaliplatin (5000 ng/mL) to maintain drug resistance. The reagents used in this study includes oxaliplatin, ferrostatin-1 and Pifithrin-α (MCE,USA), baicalin(nanjing digeer,china),MDA (solaibao,China), MitoTracker Red CM-H2 (solaibao, China), GSH detection kit, and ROS detection kit (solaibao, China). Total Iron Ion Detection Kit (Applygen,Beijing,china), doxorubicin(solaibao, China),5-fluorouracil(ZhuoTai, China),vincristine(WanLe, China).. The antibodies used includes GAPDH (NO.B1501) , IERB2 (NO.B0702) , TfR (NO.B7401) , all purchased from Inmunoway, China. GPX4 (NO.78P5366) , FTH1 (NO.32J4044) purchased from Affinity, China. The antibody concentrations used were all 1:1000.

### In vitro* cytotoxicity assay*

A total of 1 × 10^5^ cells/mL were seeded in 96-well plates. The cells were subsequently treated with baicalin and Oxaliplatin, Pifithrin-α, or Fer-1 of various concentrations. The Cell Counting Kit-8(CCK8) assay(New Cell & Molecular Biotech,SuZhou,China) was used to determine the cytotoxicity. Inhibition rate = (1 -A sample/A control) × 100% (A, absorbance).Each dose of the compound was tested in quadruplicate.

### MDA,GSH , total iron ions and ROS assays

The levels of GSH, MDA and total iron ions were measured using the respective detection kits. The cell absorbance value was measured with a spectrophotometer (SpectraMaxi3x, USA). The cellular ROS level was assessed using the ROS detection kit according to the manufacturer’s protocol. The Cells’ fluorescence intensity was measured by flow cytometry (Beckman-Coulter). Experiments were performed in triplicate.

### Mitochondrial membrane potential measurement

Baicalin and Fer-1 were used to treat HGC27 and HGC27/L cells in a six-well plate for 24 h, respectively. After collecting the cells, the fluorescent probe JC-1 was added to stain the cells. Once the cells were fully oscillated and mixed, and then observed and photographed by confocal microscope (Necope900, China). The change in mitochondrial membrane potential was measured by the ratio of red and green fluorescence of JC-1 polymer/monomer.

### Western blot assay

The protein expression was detected by western blot method. The cells were grown in 6-well plates, and a control group with no intervention, were then lyzed with RIPA buffer (Solaibao, China). The protein concentration was determined by using the BCA kit (Solaibao, China). Protein samples (20 μg) were separated on SDS-PAGE gel using a vertical electrophoresis apparatus (BIO-RAD, USA) and then transferred to a PVDF membrane (Thermo Sciences, USA). The PVDF membrane is then blocked with 10% skimmed milk and incubated with the antibody. The protein signal is detected by the ChemiDoc XRS + imaging system (Bio-Rad ,USA), using ECL reagents (New Cell & Molecular Biotech,suzhou,china), then quantified by Image Lab software. All experiments were repeated three times.

### Organelles Observation by transmission electron microscopy

The HGC27 and HGC27/L cells were planted in 6-well plates, and cultured until logarithmic growth phase. In the following steps, the cells were treated with baicalin of different concentrations (200,250,300 uM ) for 24 h, and the cells of all groups were collected, centrifuged and then prepared cell suspension. Subsequently, all cells were fixed with electron microscope fixation solution (solaibao, China), and observed and photographed with transmission electron microscope (Japan Electron 1400).

### Statistical analysis

The GraphPad Prism (version 8.0) software was used for statistical analysis and processing. Measurement data were represented as mean ± standard deviation (SD). Comparisons between groups were performed by independent sample t test. Differences between different treatment groups were analyzed using one-way analysis of variance (one-way ANOVA). *p* < 0.05 indicated statistical significance.

## Results

### Baicalin induces ferroptosis-associated cell death in drug-resistant HGC27/L gastric cancer cells

**Figure 1:**
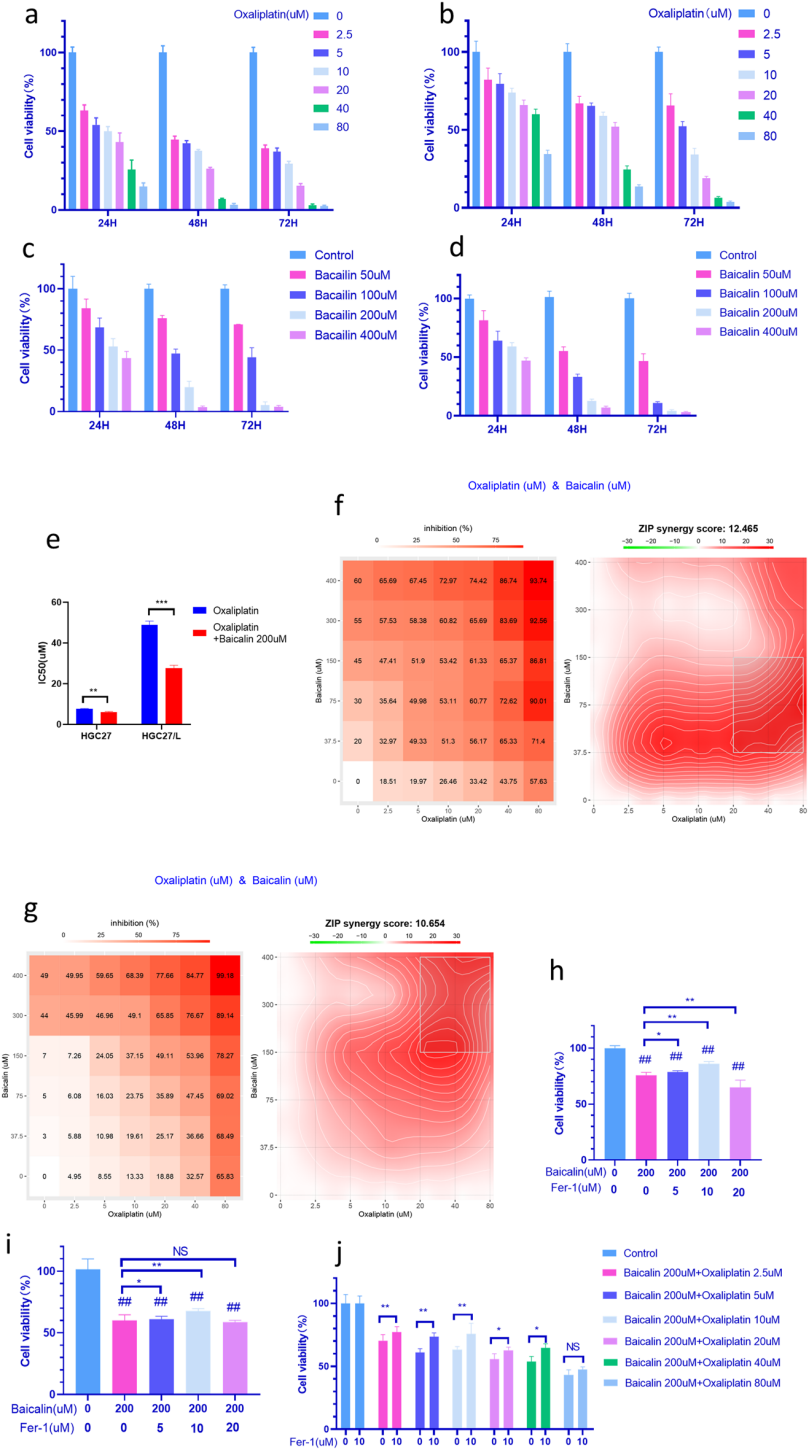
Baicalin induces -related proliferation inhibition in parental and oxaliplatin resistant gastric cancer cells. The cell viability was determined by CCK8 method after oxaliplatin treatment on HGC27 (**a**) and HGC27/L (**b**) cells, and baicalin treatment on HGC27 (**c**) and HGC27/L (**d**) for 24, 48 and 72 h, respectively. IC50 comparison between oxaliplatin alone and baicalin combined with oxaliplatin treatment on HGC27 and HGC27/L for 24 h (**e**), Oxaliplatin combined with baicalin in treatment on HGC27 (**f**) and HGC27/L (G)24 h synergistic effect diagram. After baicalin of 200 μM combined with Fer-1 intervention in HGC27 (**h**) and HGC27/L (**i**) cells for 24 h, the cell viability was determined by CCK8 method. After HGC27/L cells were treated with baicalin 200 μM combined with oxaliplatin at different concentrations in the presence or absence of Fer-1 for 24 h, the cell viability was detected by CCK8 method(**j**). *p < 0.05, **p < 0.01, **p < 0.001, ns, no statistical significance. Data are expressed as mean-standard deviation. Fer-1: ferrostatin-1. IC50: half-maximal inhibition concentration. CCK8 method: Cell Counting Kit-8. All experiments were repeated three times.

To determine the cytotoxic and chemotherapy-sensitizing effects of baicalin on HGC27/L and HGC27 gastric cancer cells, which were treated with different concentrations of baicalin and with baicalin combined with oxaliplatin. The cell viability was examined by CCK-8 assays according to the reagent manufacturer's instructions. Compared with the control group, the oxaliplatin treatment group inhibited the growth of parental HGC27 and drug-resistant HGC27/L cells in a time and dose-dependent manner significantly (Fig. 1a, b). The maximum half inhibitory concentration (IC50) of oxaliplatin for parent HGC27 and drug-resistant HGC27/L after 24 h of treatment were 7.84 μM and 47.64 μM, respectively, and the drug resistance multiple was 6.07. Baicalin at concentrations of 50, 100, 200 and 400 μM were used to treat the parental and drug-resistant cells for 24 h, 48 h and 72 h, respectively. Baicalin also inhibited the viability of parental HGC27 and drug-resistant HGC27/L cells in a time and dose-dependent manner significantly (Fig. 1c, d). The IC50 values of parental HGC27 and drug-resistant HGC27/L after 24 h of treatment were 263.6 μM and 324.7 μM, respectively. The sensitivity of HGC27/L to baicalin was lower than that of parental HGC27 cells. Next, baicalin combined with oxaliplatin to treat parental HGC27 cells and HGC27/L drug-resistant cells for 24 h to explore whether the combination of the two drugs caused a synergistic effect or not. Firstly, the IC50 values were compared after the intervention of oxaliplatin alone and oxaliplatin combined with baicalin 200 μM in parental and drug-resistant cells. The IC50 values of the combined drug group were lower than those of the single drug group with statistical differences (Fig. 1e).

The Synergy Finder software(synergyfinder 3.0)(https://synergyfinder.org/#!/) was further used to evaluate the synergistic effect by HSA model. Synergies are assessed on the basis of ZIP scores, indicating synergies when the score exceeds 10^19^. As can be seen in the figure, the ZIP score of the parent strain was 12.465, and the ZIP score of the drug-resistant strain was 10.654. The results showed that oxaliplatin combined with baicalin had synergistic anti-tumor effects on both HGC27 and HGC27/L (Fig. 1f, g).To further explore the chemo-sensitizing effect of baicalin combined with multiple chemotherapy drugs on HGC27/L and its impact on other gastric cancer cells MKN45, baicalin at 200 μM was used in combination with different concentrations (0.01, 0.1, 1, 10, 100 times the plasma peak concentration) of vincristine(VCR), doxorubicin(ADM), and 5-fluorouracil(5-FU) to intervene in HGC27/L and MKN45 cells for 24 h. The results showed that the inhibitory rate of HGC27/L and MKN45 cells was significantly higher when baicalin was used in combination compared to the intervention group treated with the same concentration of VCR, ADM, and 5-FU alone. This suggests that baicalin enhances the chemo-sensitivity of gastric cancer HGC27/L and MKN45 cells to multiple chemotherapy drugs. As shown in Tables 1 and 2 .

**Table 1:** The survival Rate of Proliferation of HGC27/L Drug-resistant Cells by baicalin Combined with VCR, ADM, and 5-FU Intervention (${\overline{\text{x}}}$  ± s, n = 6, %).

Groups	Control	0.01 × PPC	0.1 × PPC	1 × PPC	10 × PPC	100 × PPC
ADM	100 ± 3.65	98.45 ± 1.79	94.48 ± 7.08	80.12 ± 2.14	16.31 ± 6.52	1.94 ± 0.33
ADM + Baicalin 200uM	100 ± 1.69	75.3 ± 3.98^a^	66.02 ± 1.48^a^	56.42 ± 2.52^a^	8.77 ± 3.05^a^	1.13 ± 0.65
5-FU	100 ± 1.96	98.06 ± 2.11	90.6 ± 3.22	79.33 ± 1.29	53.88 ± 2.99	48.92 ± 2.51
5-FU + Baicalin 200uM	100 ± 2.19	69.73 ± 2.15^a^	53.32 ± 0.36^a^	49.24 ± 0.85^a^	32.27 ± 1.89^a^	29.3 ± 1.85^a^
VCR	100 ± 0.71	98.64 ± 1.02	93.84 ± 1.46	88.34 ± 3.49	86.89 ± 2.14	25.65 ± 3
VCR + Baicalin 200uM	100 ± 1.68	66.87 ± 1.41^a^	51.3 ± 1.06^a^	38.43 ± 0.93^a^	28.33 ± 0.81^a^	6.52 ± 0.62^a^

Compared with single VCR, ADM, 5-FU at the same concentration.

^a^*P* < 0. 01.

**Table 2:** The survival Rate of Proliferation of MKN45 gastric Cells by Baicalin Combined with VCR, ADM, and 5-FU Intervention (${\overline{\text{x}}}$ ± s, n = 6, %).

Groups	Control	0.01 × PPC	0.1 × PPC	1 × PPC	10 × PPC	100 × PPC
ADM	100 ± 4.52	96.14 ± 10.28	87.44 ± 5.77	63.66 ± 2.46	67.11 ± 5.51	47.61 ± 3.51
ADM + Baicalin 200uM	100 ± 3.67	63.62 ± 3.93^a^	61.24 ± 4.38^a^	56.25 ± 1.88^a^	45.64 ± 3.21^a^	29.83 ± 5.17^a^
5-FU	100 ± 1.56	84.38 ± 7.24	78.89 ± 3.74	75.55 ± 1.91	69.43 ± 1.7	39.68 ± 3.17
5-FU + Baicalin 200uM	100 ± 2.14	65.06 ± 8.78^a^	58.84 ± 2.26^a^	56.58 ± 3.11^a^	49.22 ± 4.12^a^	33.79 ± 3.67^a^
VCR	100 ± 1.54	72.93 ± 2.15	50.73 ± 2.36	41.7 ± 1.78	36.37 ± 2.59	24.13 ± 3.49
VCR + Baicalin 200uM	100 ± 3.49	56.15 ± 3.2^a^	38.63 ± 1.18^a^	34 ± 2.71^a^	27.96 ± 3.29^a^	15.91 ± 2.21^a^

Compared with single VCR, ADM, 5-FU at the same concentration.

^a^*P* < 0. 01.

In order to investigate whether the anti-tumor effect of baicalin is related to ferroptosis or not, baicalin of 200 μM combined with ferroptosis inhibitor Fer-1 was used to treat HGC27 and HGC27/L at different concentrations for 24 h to detect cell viability using CCK8 assay. The results showed that compared with the control group, there was a statistically significant decrease in cell viability in both the baicalin group alone and the baicalin in combination with Fer-1 group. The cell survival rate of baicalin of 200 μM combined with Fer-1 of 5 μM and Fer-1 of 10 μM groups increased compared with baicalin of 200 μM, and the survival rate of Fer-1 of 10 μM group was higher. The cell viability of the group treated with 20 μM Fer-1 combined with Baicalin was lower than that of the baicalin-alone group, which may be related to other cell death pathways caused by Fer-1 with relative higher concentrations(Fig. 1h, i). According to the experimental evidence, the anti-tumor effect of baicalin may be related to ferroptosis, and Fer-1 of 10 μM was selected for further experiments. Finally, baicalin of 200 μM was selected to combine with oxaliplatin of different concentrations in the presence or absence of Fer-1 10 μM to detect cell viability further. Similarly, it was found that Fer-1 partially reversed the cell death induced by the combination of baicalin and oxaliplatin(Fig. 1j)**,** which suggested that the anti-tumor mechanism of baicalin may be related to the activation of ferroptosis. The above experimental results indicate that baicalin has anti-tumor effects and could improve the chemotherapy sensitivity of oxaliplatin resistant gastric cancer cells when combined with oxaliplatin. The underlying mechanism may be related to baicalin's induction of ferroptosis. Therefore, ferroptosis may play a key role in baicalin-induced cell death or resistance regression.

### Baicalin activates ferroptosis in gastric cancer HGC27/L cells.

**Figure 2:**
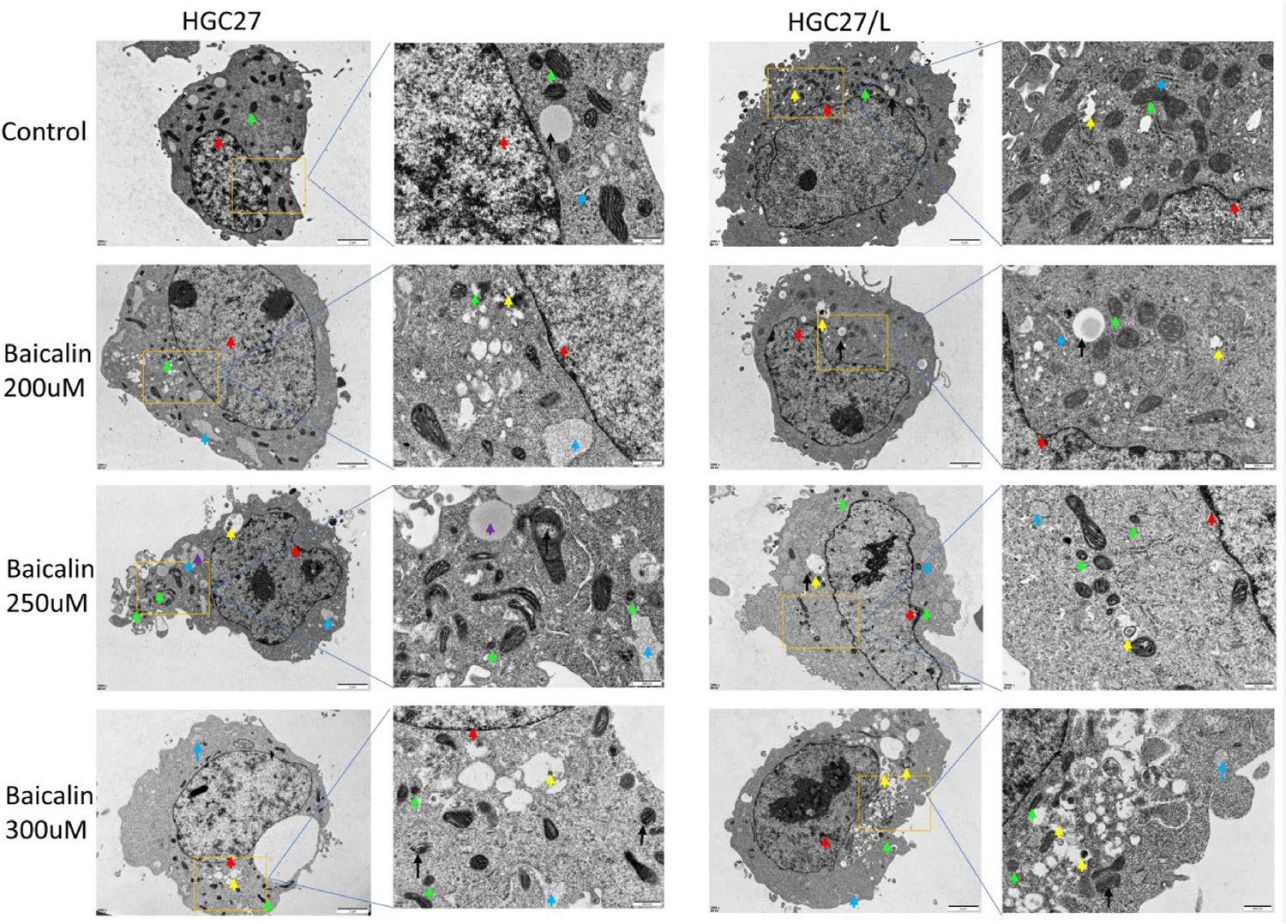
The structural changes of HGC27 and HGC27/L cells after baicalin intervention for 24 h were observed by projection electron microscopy. Nucleus, as shown by the red arrow; Intracytoplasmic linear granules, as shown by green arrows; The endoplasmic reticulum, as shown by the blue arrow; Monolayer lipid structure, as shown by the black arrow; Autophagosome, as shown by the yellow arrow. (Original magnification 200 × and 5000x).

As mitochondria play a pivotal role in cysteine-deprivation induced ferroptosis^20^ and in order to demonstrate the role of ferroptosis in baicalin treatment further, the effects of baicalin on cell ultrastructure were observed by transmission electron microscopy (Fig. 2), After HGC27 and HGC27/L were treated with baicalin of 200 μM, 250 μM and 300 μM for 24 h. In the HGC27 control group, the nuclei in the visual field was regular in shape, smooth in surface, clear in double nuclear membrane structure, normal in perinuclear space, and no agglutination was observed in nuclear staining, as shown by the red arrow. The mitochondria in the cytoplasm are abundant and complete in structure. The cristae structure in the mitochondria is clear, the arrangement is regular, and the double-layer mitochondrial membrane is normal, as shown by the green arrow. The structure of the endoplasmic reticulum is normal, with no obvious expansion, as shown by the blue arrow; A small number of monolayer lipid structures can be seen, as shown by the black arrow. After HGC27 group were treated with baicalin of 200 μM, the nuclear morphology was regular, the surface was smooth, the double nuclear membrane structure was clear, the perinuclear space was normal, and no agglutination was observed in the nuclear staining. The number of mitochondria in the cytoplasm decreased obviously, some mitochondria atrophied and the double-layer mitochondrial membrane ruptured. A small number of autophagosomes were found, which contained incomplete degraded organelles. Part of the endoplasmic reticulum was obviously dilated. When baicalin increased to 250 μM and 300 μM, irregular nuclear morphology and surface roughness were observed, the number of mitochondria in the cytoplasm was significantly reduced, some mitochondria atrophied, the double-layer mitochondrial membrane was ruptured, the mitochondrial cristae was significantly reduced, and autophagy of some mitochondria and a small number of autophagosomes were observed, which contained incompletely degraded organelles. Part of the endoplasmic reticulum was obviously dilated. In the HGC27/L control group, the nuclear morphology was irregular, the surface was uneven, the double nuclear membrane structure was clear, the perinuclear space was normal, and no agglutination was observed in the nuclear staining. The mitochondria in the cytoplasm were abundant, the structure was complete, the ridge structure in the mitochondria was clear, the arrangement was regular, and the double mitochondrial membrane was normal. A small number of autophagosomes with low monolayer homogeneity were observed. The structure of endoplasmic reticulum was normal with no obvious expansion. A few monolayer lipid structures were observed. In the drug-resistant HGC27/L cells, with the increase of the concentration of baicalin, the nuclear morphology was irregular, the surface was uneven, the double nuclear membrane structure was clear, the perinuclear space was normal, and no agglutination was observed in the nuclear staining. In the cytoplasm, a large number of mitochondria exhibited atrophy, with ruptured double-layer membranes, significantly decreased ridge structures, and increased membrane density, and a small amount of mitochondrial autophagy was observed. Some endoplasmic reticulum was slightly dilated. Many autophagosomes were observed, which contained organelles that were incompletely degraded.

After baicalin treatment, gastric cancer cells showed mitochondrial atrophy, double-layer mitochondrial membrane rupture, and the ridge structure in mitochondria decreased significantly, which was consistent with the characteristics of ferroptosis activation. In addition, the experiments showed that endoplasmic reticulum stress(ERS) appeared, and ER expansion was a typical manifestation of ERS. The occurrence of autophagosomes may be due to the regulation of autophagy in gastric cancer cells by baicalin. The results of electron microscopy showed that baicalin might play an antitumor role by activating ferroptosis and ERS in drug-resistant HGC27/L gastric cancer cells.

### Baicalin interferes with iron homeostasis of gastric cancer cells and inhibits antioxidant defenses to induce ferroptosis

**Figure 3:**
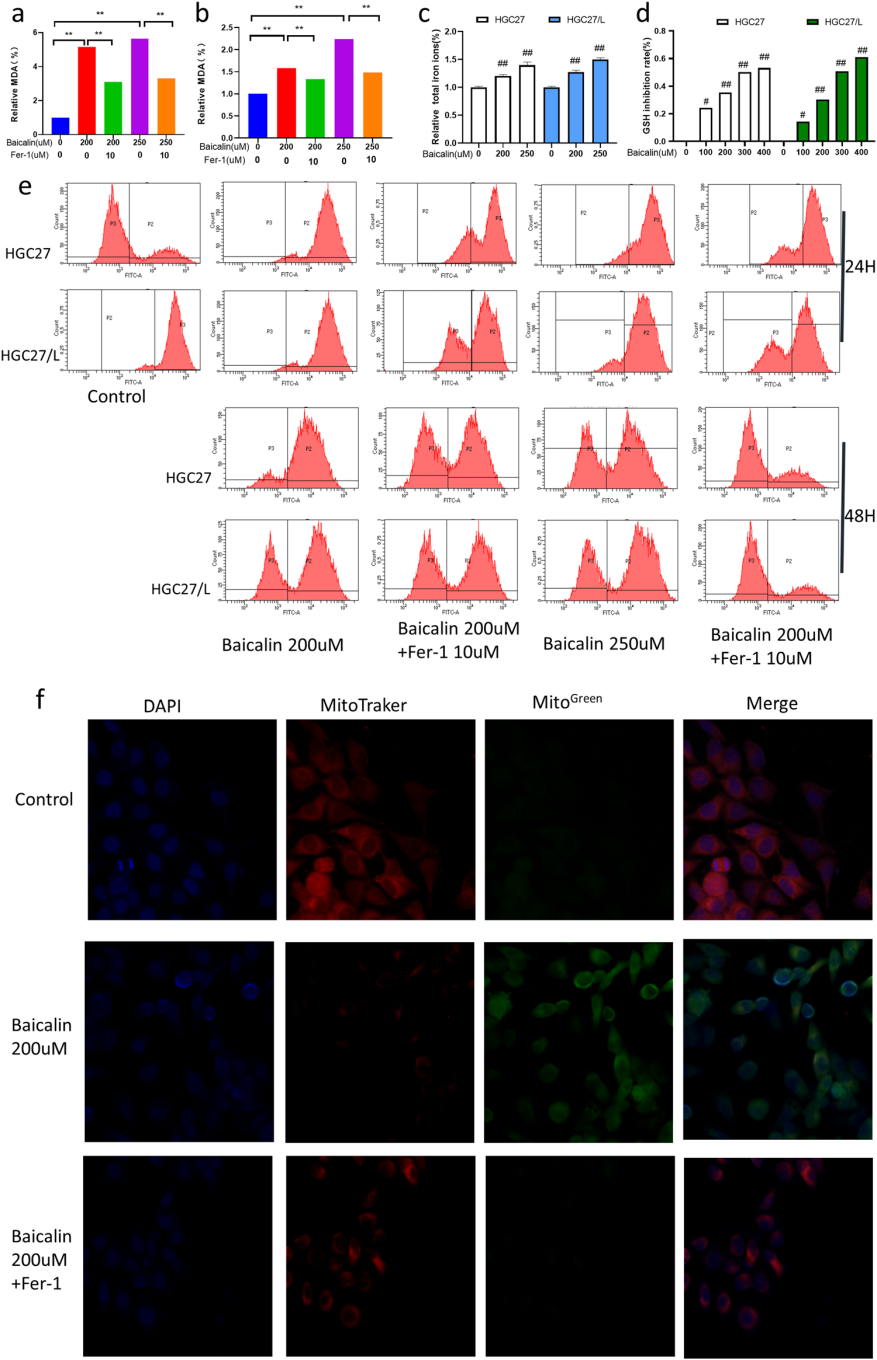
Effects of baicalin and Fer-1 on MDA content, total iron ion concentration, GSH inhibition rate, ROS accumulation and mitochondrial membrane potential after treatment on HGC27 and HGC27/L cells. The relative expression of MDA in HGC27 (**a**) and HGC27/L (**b**) cells was treated with baicalin at 200 μM, 250 μM and combined with Fer-1 for 24 h. The changes of total iron ion (**c**) and GSH inhibition rate (**d**) after baicalin treatment with 200 μM and 250 μM for 24 h in parents and resistant cells. The cumulative changes of ROS in HGC27 and HGC27/L cells were treated with baicalin at 200uM, 250uM and combined with Fer-1 after 24 h and 48 h (**e**). After the drug-resistant cells were treated with baicalin 200 μM combined with Fer-1 for 24 h, the changes of mitochondrial membrane potential were observed by confocal microscopy (original magnification 600 times) (**f**). *p < 0.05, **p < 0.01, **p < 0.001, ns had no statistical significance. Compared with the control group, #*p* < 0.05, ##*p* < 0.01, ###*p* < 0.001, ns had no statistical significance. GSH, glutathione;MDA, malondialdehyde; MMP, mitochondrial membrane potential; ROS, reactive oxygen species. All experiments were repeated three times.

**Figure 4:**
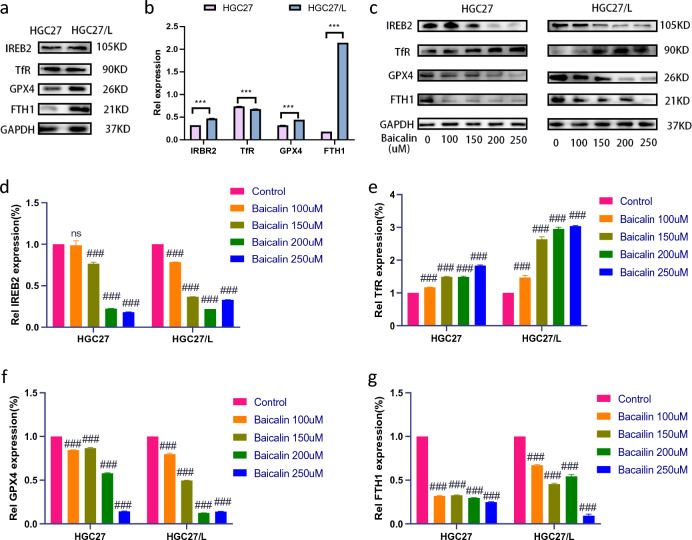
Differences of iron homeostasis and ferroptosis key proteins between HGC27 and HGC27/L cells, and the changes of that on HGC27 and HGC27/L cells after baicalin treatment for 24 h. The expressions of IRP2, FTH1, GPX4 and TfR were detected by Western blotting. Comparison of these key proteins between HGC27 and HGC27/L cells (**a**, **b**). The expressions of these key proteins (**c**–**g**) induced by baicalin was 100% in untreated cells. Representational images and quantitative bar charts are displayed. Significant differences between groups are indicated with *p < 0.05, **p < 0.01, and ***p < 0.001, ns, not significant. Compared with the control group #*p* < 0.05, ##*p* < 0.01, ###*p* < 0.001, ns had no statistical significance.Data are presented as the mean ± standard deviation. FTH1, ferritin heavy chain 1; GPX4, peroxidase-4; IRP2, iron-regulatory protein 2; TfR, transferrin receptor. Experiments were performed in triplicate.

Ferroptosis is an iron-dependent form of regulated cell death (RCD). Iron metabolism and lipid peroxidation signaling pathway are considered to be the central link in ferroptosis. Excessive iron produces ROS via Fenton reaction, resulting in ferroptosis. Circulating iron exists in the form of trivalent iron (Fe^3+^). Fe^3+^ is introduced into the nucleus by the membrane protein transferrin receptor 1 (TFR1) and reduced to ferrous iron (Fe^2+^). Finally, Fe^2+^ is released from the endosome into an unstable iron pool in the cytoplasm. When ferroptosis is induced, TFR1 expression increases and ferritin (FTL and FTH1) expression decreases. In addition to iron-mediated ROS production via Fenton reaction, GSH depletion leads to the inactivation of GPX4 , resulting in ferroptosis resulting from the production of ROS by lipid peroxidation. In addition, mitochondria play an important role in ROS production and regulation of cell death^20–22^. Therefore, after treating with different concentrations of baicalin, peroxidase malondialdehyde (MDA) content (Fig. 3a, b), total iron ion concentration (Fig. 3c) and GSH inhibition rate (Fig. 3d) in parental HGC-27 and drug-resistant cells HGC-27/L were detected by colorimetry,.Additionally, ROS production (Fig. 3e) was detected by flow cytometry. The changes of mitochondrial membrane potential were observed by confocal microscopy (Fig. 3f). The expressions of ferroptosis-related genes were detected by WB method (Fig. 4). The results showed that compared with baicalin alone group (Fig. 3a, b), baicalin of 200 μM and 250 μM could significantly induce MDA production in the two cell lines after intervention for 24 h in the presence or absence of Fer-1. When baicalin combined with Fer-1 of 10 μM was administrated on HGC-27 and HGC-27/L cells, MDA production was partially inhibited. Compared with the control group, the total iron ion concentration (Fig. 3c) and GSH inhibition rate (Fig. 3d) results showed a significantly dose-dependent increase after baicalin treatment for 24 h. Subsequently, the ROS production rates of each group were detected by flow cytometry after 24 and 48 h of treatment with baicalin of 200 μM and 250 μM in the presence or absence of Fer-1 of 10 μM. The results showed that under the same exposure time, baicalin induced ROS production in the two groups in a dose-dependent manner, and the ROS production rate was partially inhibited after the combination of Fer-1 (Fig. 3e). Ferroptosis induced mitochondrial membrane potential hyperpolarization and lipid peroxidation accumulation. Therefore, the mitochondrial membrane potential changes of gastric cancer resistant cells HGC27/L were examined after 24 h intervention by baicalin of 200 μM alone or combined with Fer-1 of 10 μM. Baicalin can lead to a decrease in HGC27/L cell membrane potential, which can be partially inhibited by ferroptosis inhibitors (Fig. 3).

To investigate the effects of iron homeostasis regulation and antioxidant defense effects on drug resistance, the expressions of TfR, FTH1, GPX4, and IREB2 were compared between HGC27 and HGC27/L cells (Fig. 4). Firstly, the expressions of TfR, FTH1, GPX4 and IREB2 in parental and drug-resistant cells were compared. It was found that HGC27/L cells had higher expressions of IREB2, FTH1, and GPX4 than HGC27 cells, and lower expression of TfR than parental HGC27 cells, which may contribute to increased buffering capacity against iron disturbances, through the production of a key protein for these ferroptosis to occur and chemotherapy drug resistance (Fig. 4a,b). Then, both HGC27 and HGC27/L cells were treated with different concentrations of baicalin for 24 h to explore the changes of key genes of ferroptosis, such as TfR, FTH1, GPX4 and IREB2 in gastric cancer cells exposed to baicalin with different concentrations. Baicalin treatment for 24 h dose-dependently decreased the expression of IREB2, FTH1, and GPX4 in both cell lines (Fig. 4c,d,g), while increased the expression of TfR, a gene responsible for iron uptake (Fig. 4e)^23^.On the whole, baicalin-induced ferroptosis may be achieved through the key genes mentioned above.

In summary, baicalin targets ferroptosis triggered by the accumulation of lipid peroxides and the production of ROS by regulating iron metabolism.

### Baicalin enhances the chemotherapy sensitivity of gastric cancer cells HGC27 / L by activating ferroptosis through upregulation of SLC7A11/GPX4/ROS mediated by p53

**Figure 5:**
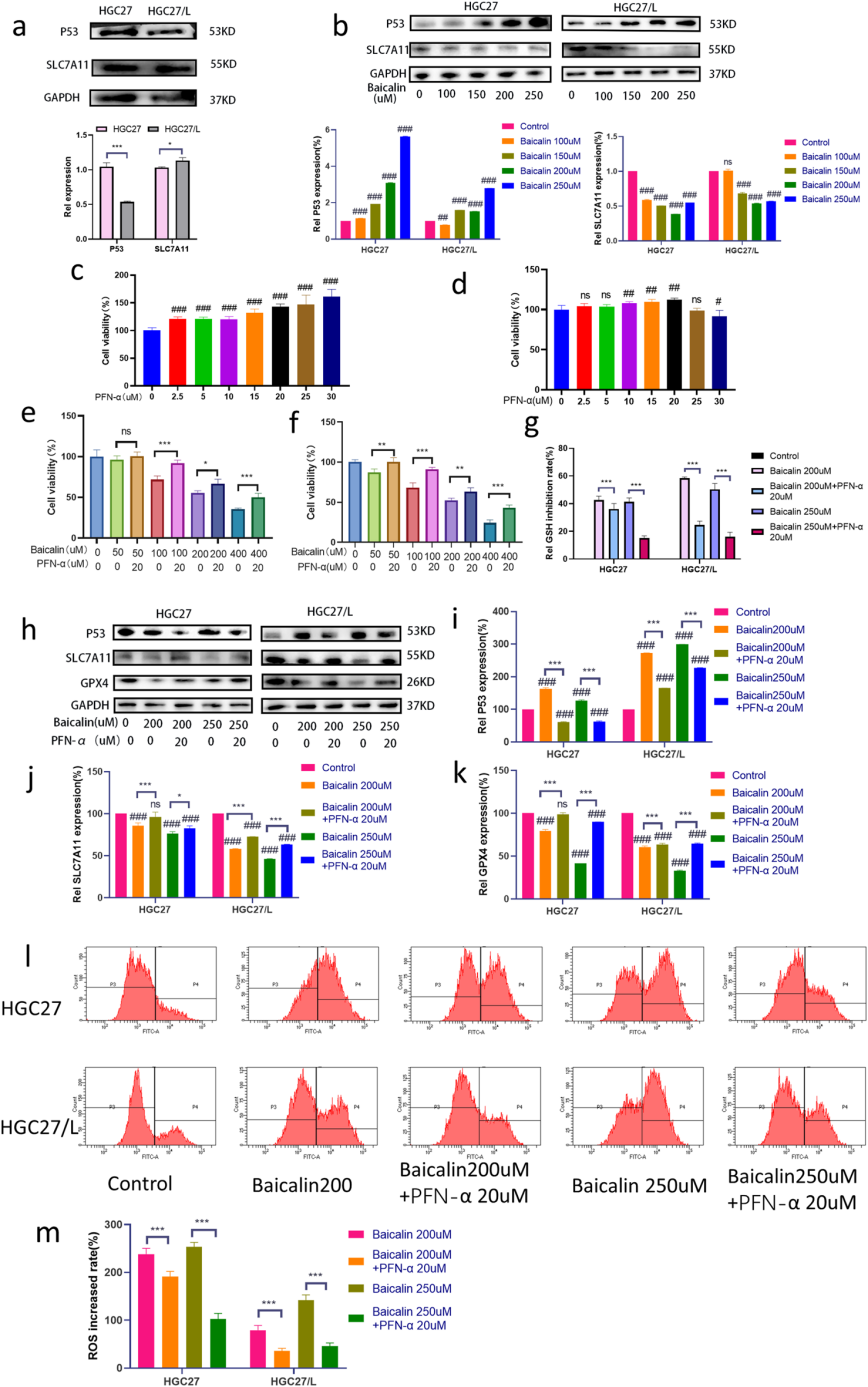
p53-mediated SLC7A11/GPX4/ROS pathway is involved in oxaliplatin resistance and death of sensitive gastric cancer cells induced by Baicalin. Western blotting was used to detect the difference of (**a**) and (**b**) genes p53 and SLC7A11 between HGC27 and HGC27/L cells, and the changes of that on HGC27 and HGC27/L cells after treated with baicalin of different concentrations. After HGC27 (**c**) and HGC27 (**d**) were treated with PFN-α of different concentrations for 24 h, The cell viability was determined by CCK8 method. After HGC27 (**e**) and HGC27/L (**f**) were treated with baicalin of different concentrations combined with PFN-α 20 μM for 24 h, the cell viability CCK8 and GSH inhibition rate was detected (**g**). After HGC27 and HGC27/L cells were treated with baicalin at 200 μM, 250 μM and combined with PFN-α of 20 μM for 24 h, the changes of genes p53, SLC7A11 and GPX4 were detected by Western blotting (h,i,j,k), and ROS accumulation was detected by flow cytometry (l, m). The expression of corresponding protein in untreated cells was 100%. Representational images and quantitative bar charts are displayed. The difference between groups was significant (*p < 0.05, **p < 0.01, *** p < 0.001, ns). Compared with the control group, #*p* < 0.05, ##*p* < 0.01, ###*p* < 0.001, ns had no statistical significance. Data are expressed as mean ± standard deviation. SLC7A11, solute carrier family 7 member 11; SLC7A11, Solute carrier family 7 member 11; GPX4, peroxisase-4. PFN-α: Pifithrin-α. The experiment was repeated three times.

P53 is a typical tumor suppressor gene, which plays an important role in the regulation of various cell metabolism and ferroptosis related genes^24^. Solute carrier family 7 member 11 (SLC7A11) acts as a cystine transporter^25^. p53 blocks cystine intake by inhibiting the transcription of SLC7A11 protein, thus limiting the synthesis of intracellular GSH, resulting in increased cell sensitivity to ferroptosis ^26^. In order to explore the potential mechanism of baicalin activation and clarify its influence on p53 gene, the differences between p53 and SLC7A11 (Fig. 5a) and GPX4 (Fig. 4a,b) in HGC27 and HGC27/L cells were compared. Compared with parental HGC27 cells, it was found that p53 expression was significantly reduced in HGC27/L cells. Then, the expressions of p53, SLC7A11 (Fig. 5b) and GPX4 (Fig. 4f) were evaluated after 24 h of baicalin intervention in the two cell lines. The results showed that baicalin significantly upregulated p53 expression and inhibited SLC7A11 and GPX4 expression in a dose-dependent manner. To further clarify the effect of baicalin on p53-mediated ferroptosis, the p53 inhibitor PFN-α was used to inhibit p53 expression in two cell lines for 24 h. When PFN-α was used to treat HGC27 (Fig. 5c) and HGC27/L (Fig. 5d), it was found that PFN-α could promote the survival of tumor cells, which was considered to be related to the inhibition of the expression of tumor suppressor gene p53 by PFN-α. In HGC27/L cells, when the concentration of PFN-α was greater than 20 μM, the cell viability was weakened. Considering the possible toxic effect of drug concentration, PFN-α of 20 μM was selected for following experimental study. Baicalin with different concentrations combined with PFN-α of 20 μM were selected to treat the two cell lines for 24 h, and the CCK8 assay detected cell viability. As can be seen in the figure, compared with baicalin treatment with the concentrations of 100, 200 and 400 μM alone in HGC27 cells, the combination of PFN-α increased cell survival (Fig. 5e). Compared with the four concentrations of baicalin in HGC27/L cells, the cell viability of the baicalin and PFN-α groups were enhanced significantly (Fig. 5f). These results suggest that baicalin may play an antitumor role by up-regulating the tumor suppressor gene p53. To explore whether baicalin could regulate the occurrence of ferroptosis in gastric cancer through p53 or not, baicalin of 200 μM and 250 μM combined with PFN-α of 10 μM were used to treat HGC27/L cells for 24 h, and the inhibition rate of GSH, the expressions of genes p53 (Fig. 5g), SLC7A11, GPX4 (Fig. 5h), and ROS changes (Fig. 5l, m) were is needed. PFN-α can weaken the inhibition of baicalin on GSH in HGC27 and HGC27/L cells (Fig. 5g). Compared with the baicalin group, p53 gene expression was inhibited in the baicalin and PFN-α groups, and SLC7A11 and GPX4 expressions were increased (Fig. 5h,i,j,k), and PFN-α could reverse part of baicalin-induced ROS accumulation. These results indicate that ferroptosis can be inhibited by inhibiting p53 expression. To sum up, p53 plays an important role in baicalin-activated ferroptosis. Baicalin could enhance the chemotherapy sensitivity of resistant gastric cancer cells HGC-27 / L by activating ferroptosis through up-regulation of SLC7A11/GPX4/ROS mediated by tumor suppressor gene p53.

## Discussion

The current treatment methods for gastric cancer include surgery, radiotherapy, chemotherapy, targeted therapy, etc., but there are some problems such as low cure rate, poor prognosis, many adverse reactions and drug resistance. Advanced gastric cancer is treated with sequential chemotherapy, in which platinum drugs are the first-line drugs. Chemotherapy resistance is the main obstacle to cure^1,27^. In order to overcome the barrier of MDR, one of the main goals of cancer treatment research is to develop effective therapeutic drugs with few side effects and overcome drug resistance. Many natural anti-tumor drugs have the mentioned-above characteristics and enter the research and application^28^. Baicalin, a kind of natural medicine isolated and purified from Scutellaria baicalensis, has attracted more and more attention in recent years because of its good anti-tumor effect. Baicalin inhibited the proliferation and migration of rectal cancer cells, induced cell cycle arrest, epithelial-mesenchymal transformation (EMT) and stemness stem to inhibit cell growth, migration and invasion, and induced cell apoptosis^29^. In another colon cancer study, baicalin was found to induce senescence of human colon cancer cells by up-regulating DEPP and activating Ras/Raf/MEK/ERK signaling^30^. In the study of lung cancer, baicalin increased the expression of miR-340-5p and induced anti-tumor activity by affecting the miR-340-5p/NET1 axis^8^. In the study of ovarian cancer, baicalin can inhibit ovarian cancer stem cell by decreasing YAP activity^9^. Baicalin could also play an antitumor role by regulating ferroptosis. Previous studies have found that ferroptosis was induced in bladder cancer cells by down-regulating FTH1^6^, and ferroptosis in osteosarcoma was induced by a novel Nrf2/xCT/GPX4 regulatory axis^31^. The mentioned-above studies indicated that baicalin can exert anti-tumor effects through multiple pathways and multiple targets.

In this work, we investigated the possibility of baicalin's involvement in the chemotherapy sensitization mechanism in oxaliplatin-resistant gastric cancer cells. Our final findings showed that baicalin can inhibit cell viability through ferroptosis. It also has a synergistic effect when combined with oxaliplatin. baicalin exhibits anti-tumor effects on drug-resistant cells HGC27/L and gastric cancer parent cells HGC27 in a time and dose-dependent manner. We aimed to enhance the sensitivity of gastric cancer resistant cells HGC27/L to chemotherapy. Subsequent research revealed the cooperative and synergistic effects of baicalin-activated ferroptosis, confirming the efficacy of Chinese medicine in treating conditions with numerous targets and pathways. Our study aims to provide theoretical support for finding new drugs and therapeutic targets to overcome multi-drug resistance in gastric cancer.

Ferroptosis, a novel kind of cell death that differs from apoptosis and is brought on by the hazardous buildup of lipid peroxides on the cell membrane, holds considerable promise for treating cancer by suppressing tumor formation and enhancing tumor immunity^32^. Chemotherapy resistance may be reversed by modifications to the signaling pathways linked to ferroptosis. Research has demonstrated that ferroptosis inhibition can increase gastric cancer's resistance to chemotherapy^17^, while ferroptosis promotion can increase the cancer's sensitivity to chemotherapy^18^. Mitochondria power life through their diverse metabolic functions. Mitochondria play an important role in the ferroptosis process^33^. Mitochondrial function influences the two major defense system functions of glutathione peroxidase 4 (GPX4) and ferroptosis suppressant protein 1 (FSP1) in ferroptosis ^34^. Inhibition of mitochondrial tricarboxylic acid cycling or electron transfer chain can reduce mitochondrial membrane potential hyperpolarization, lipid peroxide accumulation and ferroptosis^20^. This study found that , the mitochondrial shrinkage, mitochondrial membrane density increased, mitochondrial ridge decreased or disappeared, and membrane potential decreased following the parent and drug-resistant cell lines of gastric cancer were treating with baicalin. The above mitochondrial changes are typical when ferroptosis occurs.

In order to promote growth, cancer cells have an increased demand for iron compared to normal non-cancer cells. This iron dependence makes cancer cells more susceptible to iron-catalyzed necrosis, and this cell death execution mechanism is often defective in drug-resistant cells^35^. Ferroptosis induction is dependent on an increase in lipid peroxidation brought on by iron buildup and the generation of free radicals. In tumor cells, there is a decrease in iron storage, an increase in iron uptake, and a restricted iron outflow. Lipid peroxidation and lipid membrane rupture are caused by the subsequent accumulation of excess iron, which also causes the iron-dependent Fenton reaction and the activation of iron-containing enzymes to produce ROS^36^. ROS regulates the proliferation, survival and drug resistance of cancer cells. ROS levels and clearance/antioxidant enzyme activity are generally increased in resistant cancer cells compared to non-MDR cancers and normal cells^37^. ROS may act as a second messenger in signal transduction systems and be sensitive to redox states^38^. Blocking of the intracellular antioxidant system is another way to induce ferroptosis^22,36^. GPX4 can detoxify membrane lipid peroxidation by reducing glutathione (GSH) as a cofactor. GSH deficiency may limit the antioxidant efficiency of GPX4. The disruption of the balance between oxidative damage and antioxidant defense shapes the process of ferroptosis ^16,39^. In this study, the experiment results showed that baicalin in HGC27 and HGC27/L cells could induce MDA, iron ion concentration, GSH inhibition rate and ROS production all increased in a dose-dependent manner in HGC27 and HGC27/L cells. MDA and ROS induced by baicalin can be partially reversed by Fer-1. While MMP decreased following baicalin intervention, similarly, MMP partially recovered after combined Fer-1 intervention. To our surprise, it is the first time that baicalin triggers ferroptosis in vitro through both the blocking of the antioxidant system and the enrichment of iron chelate in cells. Mechanistically, we found that ROS is a key mediator of baicalin-induced iron sag in bladder cancer cells.

Increased iron uptake and decreased iron storage can lead to iron overload during iron metabolism. Several key proteins are involved in iron uptake and storage, including the transferrin receptor (TfR), which is thought to be involved in ferric ion uptake, whose expression increases susceptibility to ferroptosis inducers^40^; Ferritin heavy chain 1(FTH1), involved in iron storage and efflux^6,41^; And iron-regulatory proteins, which translate and control a group of iron metabolism genes, including TfR and FTH1, by binding to iron response element binding protein 2 (IREB2)^36,42^. The degradation of ferritin increases the iron levels of the cells, leading to the accumulation of ROS, which ultimately leads to cell death^43^. In this study, we observed that drug-resistant cells exhibited lower expression of transferrin receptor (TfR) and higher expression of ferritin heavy chain 1 (FTH1), iron-regulatory protein 2 (IREB2), and glutathione peroxidase 4 (GPX4) compared with their parental cell line, with a significant upregulation of FTH1. The expression level of TfR is typically proportional to the iron demand of cells, participating in iron absorption to meet the requirements of iron-dependent metabolism. On the other hand, FTH1 regulates intracellular iron homeostasis by enhancing iron storage and release, which may contribute to alleviating iron metabolism disorders and potentially increasing cell drug resistance.^44^. Following the intervention with baicalin, the expression of FTH1, IREB2, and GPX4 decreased in a dose-dependent manner in both HGC27 and HGC27/L cells, while the expression of TfR increased. Baicalin modulated the levels of these proteins, resulting in increased iron absorption, decreased iron storage, and altered iron release, leading to iron accumulation in HGC27 and HGC27/L cells. Subsequently, excessive iron promoted the generation of reactive oxygen species (ROS) through iron-dependent Fenton reactions and the activation of iron-containing enzymes, which further induced lipid peroxidation and lipid membrane rupture, triggering and activating ferroptosis, thereby enhancing chemotherapy sensitivity.p53 is an important tumor suppressor gene, and direct or indirect intervention with p53 has a good anti-tumor effect^45^. p53 mutations also play an important role in the resistance of cancer cells to chemotherapy drugs^46^. In the study of cisplatin-resistant gastric cancer, it was found that exosome transmitted miR-769-5p conferred cisplatin resistance and progression in gastric cancer by targeting CASP9 and promoting ubiquitination degradation of p53^47^. In colorectal cancer studies with cisplatin resistance, mismatch repair defects and p53-mediated changes in DNA damage signaling are the main factors regulating drug resistance ^48^. p53 inhibits cystine uptake by inhibiting the expression of SLC7A11, a key component of the cystine/glutamate reverse transporter, and sensitizes cells to ferroptosis under reactive oxygen species (ROS) -induced stress^26^. Cysteine is the rate-limiting precursor of glutathione synthesis, and intracellular cysteine is mainly provided by SLC7A11-mediated cystine uptake. Ferroptosis was induced in many cancer cells when cystine was removed from cell culture or when SLC7A11 was inactivated by gene ablation or drug inhibition. In contrast, SLC7A11 overexpression in cancer cells promotes glutathione biosynthesis and is insensitive to ferroptosis^25,49^.

This study discovered that drug-resistant strains had lower p53 gene expression than parental strains, and that baicalin intervention could greatly increase p53 expression. Simultaneously, it was found that baicalin intervention could inhibit SLC7A11 and GPX4 in the p53-mediated SLC7A11/GPX4/ROS pathway, and ROS increased cumulatively, thereby targeting the activation of ferroptosis. The application of Pifithrin-α, an inhibitor of p53 post-transcriptional activity^50^ , to HGC27 and HGC27/L cells counteracted some of the effects of baicalin on SLC7A11 and GPX4, as well as the build-up of reactive oxygen species. The aforementioned experimental findings demonstrate that baicalin is a useful intervention medication and that the p53-mediated SLC7A11/GPX4/ROS pathway is an excellent therapeutic target for reversing oxaliplatin-resistant gastric cancer.

In summary, the results of all in vitro experiments showed that baicalin can play an anti-tumor role in gastric cancer and enhance the sensitivity of oxaliplatin resistance to HGC27/L through multiple pathways and multiple targets. Its mechanism mainly includes interfering with iron homeostasis and inhibiting antioxidant defense, up-regulating the tumor suppressor gene p53 to induce ferroptosis.

### Supplementary Information

Supplementary Information.

## Data Availability

The data that support the findings of this study are available on request from the corresponding author. The data are not publicly available due to privacy or ethical restrictions.

## References

[1] Wei L (2020). Noncoding RNAs in gastric cancer: implications for drug resistance. Molecular cancer.

[2] Cao T (2022). A CGA/EGFR/GATA2 positive feedback circuit confers chemoresistance in gastric cancer. J. Clin. Investigat..

[3] Kim R (2022). Early tumor-immune microenvironmental remodeling and response to first-line fluoropyrimidine and platinum chemotherapy in advanced gastric cancer. Cancer discovery.

[4] Li B (2020). Efficiency of Traditional Chinese medicine targeting the Nrf2/HO-1 signaling pathway. Biomedicine Pharmacotherapy.

[5] Hu Q (2021). Baicalin and the liver-gut system: Pharmacological bases explaining its therapeutic effects. Pharmacological research.

[6] Kong N (2021). Baicalin induces ferroptosis in bladder cancer cells by downregulating FTH1. Acta pharmaceutica Sinica. B.

[7] Yuan J (2023). Baicalin enhances the efficacy of 5-Fluorouracil in gastric cancer by promoting ROS-mediated ferroptosis. Biomedicine pharmacotherapy.

[8] Zhao F (2021). Baicalin suppresses lung cancer growth phenotypes via miR-340-5p/NET1 axis. Bioengineered.

[9] Li Y (2020). Baicalin attenuates YAP activity to suppress ovarian cancer stemness. OncoTargets and therapy.

[10] Jiang H (2022). Baicalin suppresses the progression of Type 2 diabetes-induced liver tumor through regulating METTL3/m(6)A/HKDC1 axis and downstream p-JAK2/STAT1/clevaged Capase3 pathway. Phytomedicine : international journal of phytotherapy and phytopharmacology.

[11] Lin MY (2021). Baicalin enhances chemosensitivity to doxorubicin in breast cancer cells via upregulation of oxidative stress-mediated mitochondria-dependent apoptosis. Antioxidants.

[12] Ganguly R, Gupta A, Pandey AK (2022). Role of baicalin as a potential therapeutic agent in hepatobiliary and gastrointestinal disorders: A review. World journal of gastroenterology.

[13] Singh S, Meena A, Luqman S (2021). Baicalin mediated regulation of key signaling pathways in cancer. Pharmacological research.

[14] Dixon SJ (2012). Ferroptosis: an iron-dependent form of nonapoptotic cell death. Cell.

[15] Stockwell BR, Jiang X, Gu W (2020). Emerging Mechanisms and Disease Relevance of Ferroptosis. Trends in cell biology.

[16] Zhang C, Liu X, Jin S, Chen Y, Guo R (2022). Ferroptosis in cancer therapy: a novel approach to reversing drug resistance. Molecular cancer.

[17] Zhang H (2020). CAF secreted miR-522 suppresses ferroptosis and promotes acquired chemo-resistance in gastric cancer. Molecular cancer.

[18] Ouyang S (2022). Inhibition of STAT3-ferroptosis negative regulatory axis suppresses tumor growth and alleviates chemoresistance in gastric cancer. Redox biology.

[19] Ianevski A, Giri AK, Aittokallio T (2022). SynergyFinder 3.0: an interactive analysis and consensus interpretation of multi-drug synergies across multiple samples. Nucleic acids research.

[20] Gao M (2019). Role of Mitochondria in Ferroptosis. Molecular cell.

[21] Tang D, Chen X, Kang R, Kroemer G (2021). Ferroptosis: molecular mechanisms and health implications. Cell research.

[22] Xie Y (2016). Ferroptosis: process and function. Cell death and differentiation.

[23] Mou Y (2019). Ferroptosis, a new form of cell death: opportunities and challenges in cancer. Journal of hematology & oncology.

[24] Gnanapradeepan K (2018). The p53 Tumor Suppressor in the Control of Metabolism and Ferroptosis. Frontiers in endocrinology.

[25] Koppula P, Zhuang L, Gan B (2021). Cystine transporter SLC7A11/xCT in cancer: ferroptosis, nutrient dependency, and cancer therapy. Protein & cell.

[26] Jiang L (2015). Ferroptosis as a p53-mediated activity during tumour suppression. Nature.

[27] Smyth EC, Nilsson M, Grabsch HI, van Grieken NC, Lordick F (2020). Gastric cancer. Lancet (London, England).

[28] Kumar A, Jaitak V (2019). Natural products as multidrug resistance modulators in cancer. European journal of medicinal chemistry.

[29] Yang B, Bai H, Sa Y, Zhu P, Liu P (2020). Inhibiting EMT, stemness and cell cycle involved in baicalin-induced growth inhibition and apoptosis in colorectal cancer cells. Journal of Cancer.

[30] Wang Z (2018). Baicalin induces cellular senescence in human colon cancer cells via upregulation of DEPP and the activation of Ras/Raf/MEK/ERK signaling. Cell death & disease.

[31] Wen RJ (2023). Baicalin induces ferroptosis in osteosarcomas through a novel Nrf2/xCT/GPX4 regulatory axis. Phytomedicine : international journal of phytotherapy and phytopharmacology.

[32] Lei G, Zhuang L, Gan B (2022). Targeting ferroptosis as a vulnerability in cancer. Nature reviews. Cancer.

[33] Bock FJ, Tait SWG (2020). Mitochondria as multifaceted regulators of cell death. Nature reviews. Molecular cell biology.

[34] Mao C (2021). DHODH-mediated ferroptosis defence is a targetable vulnerability in cancer. Nature.

[35] Hassannia B, Vandenabeele P, Vanden Berghe T (2019). Targeting ferroptosis to iron out cancer. Cancer cell.

[36] Chen X, Kang R, Kroemer G, Tang D (2021). Broadening horizons: the role of ferroptosis in cancer. Nature reviews. Clinical oncology.

[37] Cui Q (2018). Modulating ROS to overcome multidrug resistance in cancer. Drug resistance updates : reviews and commentaries in antimicrobial and anticancer chemotherapy.

[38] Madreiter-Sokolowski CT, Thomas C, Ristow M (2020). Interrelation between ROS and Ca(2+) in aging and age-related diseases. Redox biology.

[39] Seibt TM, Proneth B, Conrad M (2019). Role of GPX4 in ferroptosis and its pharmacological implication. Free radical biology & medicine.

[40] Hiromatsu M (2023). Transferrin Receptor is Associated with Sensitivity to Ferroptosis Inducers in Hepatocellular Carcinoma. Annals of surgical oncology.

[41] Zhang R (2022). Curcumenol triggered ferroptosis in lung cancer cells via lncRNA H19/miR-19b-3p/FTH1 axis. Bioactive materials.

[42] Wang H (2020). FBXL5 Regulates IRP2 Stability in Iron Homeostasis via an Oxygen-Responsive [2Fe2S] Cluster. Molecular cell.

[43] Park E, Chung SW (2019). ROS-mediated autophagy increases intracellular iron levels and ferroptosis by ferritin and transferrin receptor regulation. Cell death & disease.

[44] Zhang X (2022). Dihydroartemisinin Triggers Ferroptosis in Multidrug-Resistant Leukemia Cells. DNA and cell biology.

[45] Hu J (2021). Targeting mutant p53 for cancer therapy: direct and indirect strategies. Journal of hematology & oncology.

[46] Cao X, Hou J, An Q, Assaraf YG, Wang X (2020). Towards the overcoming of anticancer drug resistance mediated by p53 mutations. Drug resistance updates : reviews and commentaries in antimicrobial and anticancer chemotherapy.

[47] Jing X (2022). Exosome-transmitted miR-769-5p confers cisplatin resistance and progression in gastric cancer by targeting CASP9 and promoting the ubiquitination degradation of p53. Clinical and translational medicine.

[48] Köberle B, Schoch S (2021). Platinum Complexes in Colorectal Cancer and Other Solid Tumors. Cancers.

[49] Ji X (2018). xCT (SLC7A11)-mediated metabolic reprogramming promotes non-small cell lung cancer progression. Oncogene.

[50] Dinca EB (2008). p53 Small-molecule inhibitor enhances temozolomide cytotoxic activity against intracranial glioblastoma xenografts. Cancer research.

