# Supplementary material for: Baicalin enhances the chemotherapy sensitivity of oxaliplatin-resistant gastric cancer cells by activating p53-mediated ferroptosis
Source: Sci Rep. 2024 May 10;14:10745. doi: 10.1038/s41598-024-60920-y (PMC11087583; doi:10.1038/s41598-024-60920-y)
Supplement: Supplementary file 1 — Supplementary Information. [file 41598_2024_60920_MOESM1_ESM.pdf]

Original image of Western bolt

1.IREB2

Reference:

Xia H, Wu Y, Zhao J, Cheng C, Lin J, Yang Y, Lu L, Xiang Q, Bian T, Liu Q. N6-Methyladenosine-modified circSAV1 triggers ferroptosis in COPD through recruiting YTHDF1 to facilitate the translation of IREB2. Cell Death Differ. 2023 May;30(5):1293-1304. doi: 10.1038/s41418-023-01138-9. Epub 2023 Feb 24. PMID: 36828914; PMCID: PMC10154389.

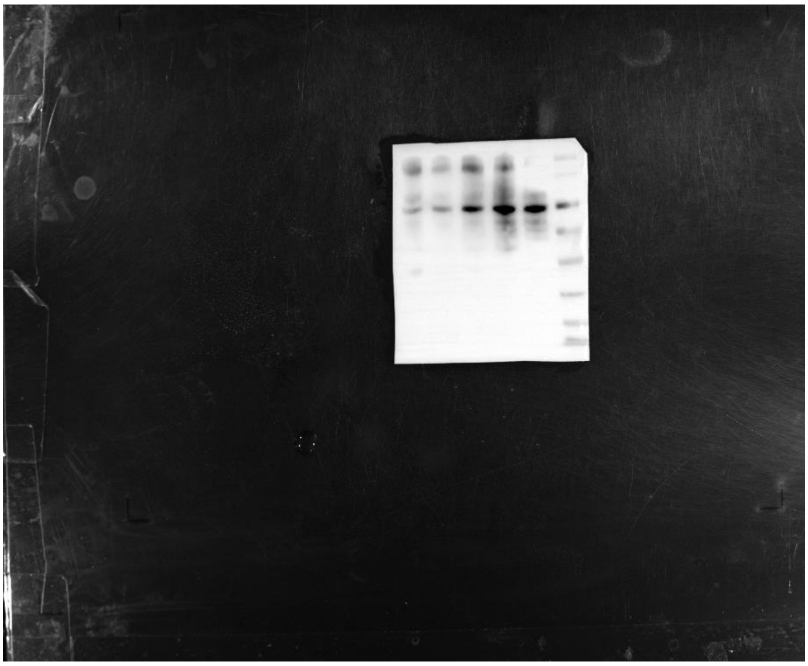

105kD

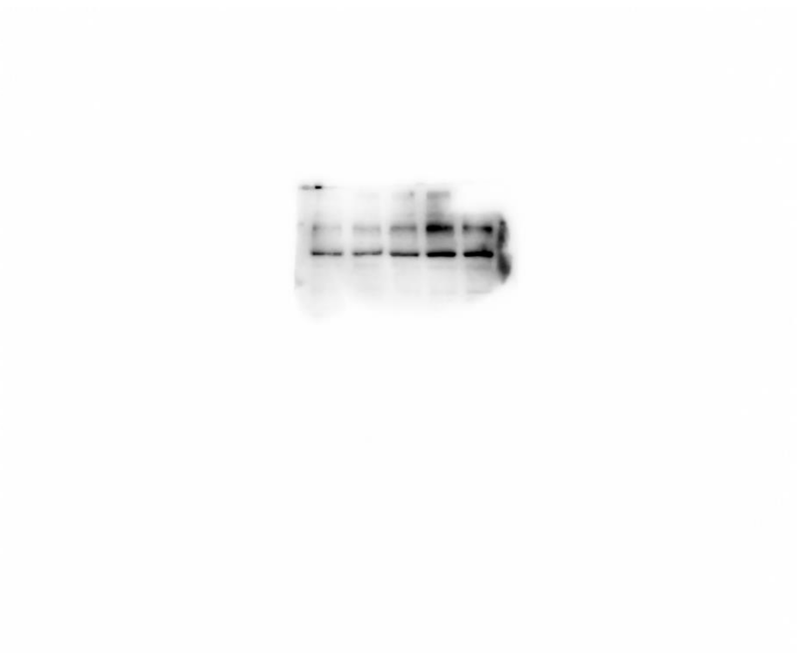

105kD

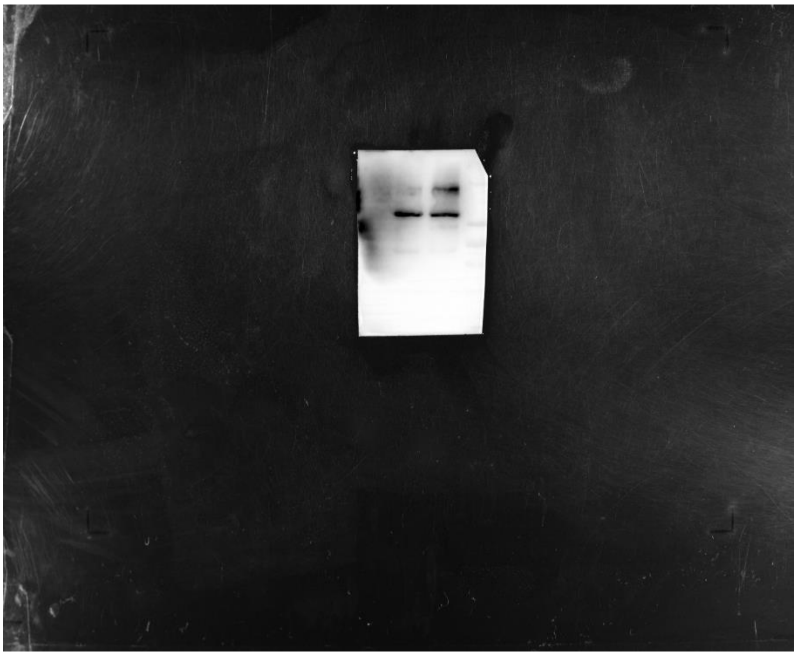

105kD

TfR

Reference:

Huang S, Wang Y, Xie S, Lai Y, Mo C, Zeng T, Kuang S, Zhou C, Zeng Z, Chen Y, Huang S, Gao L, Lv Z. Isoliquiritigenin alleviates liver fibrosis through caveolin-1-mediated hepatic stellate cells ferroptosis in zebrafish and mice. Phytomedicine. 2022 Jul;101:154117. doi: 10.1016/j.phymed.2022.154117. Epub 2022 Apr 20. PMID: 35489326.

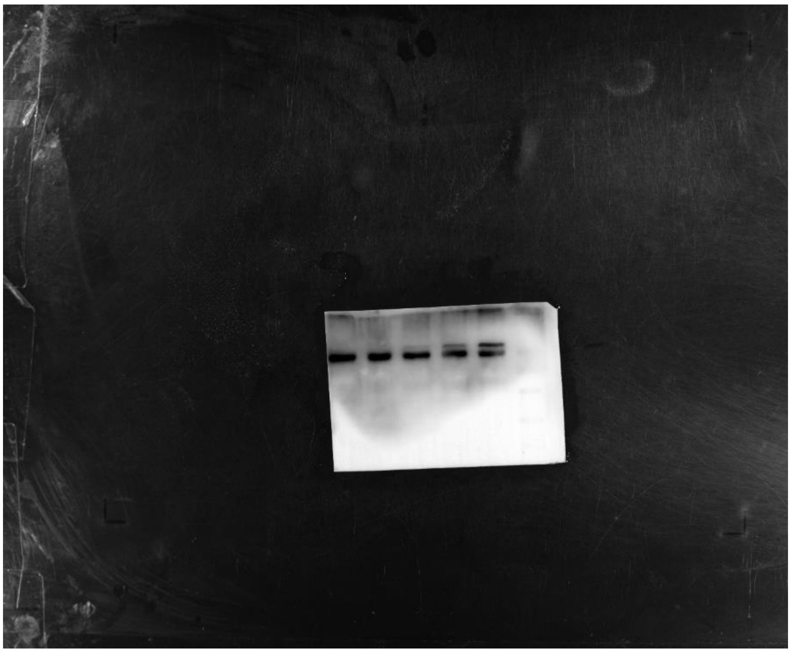

90kD

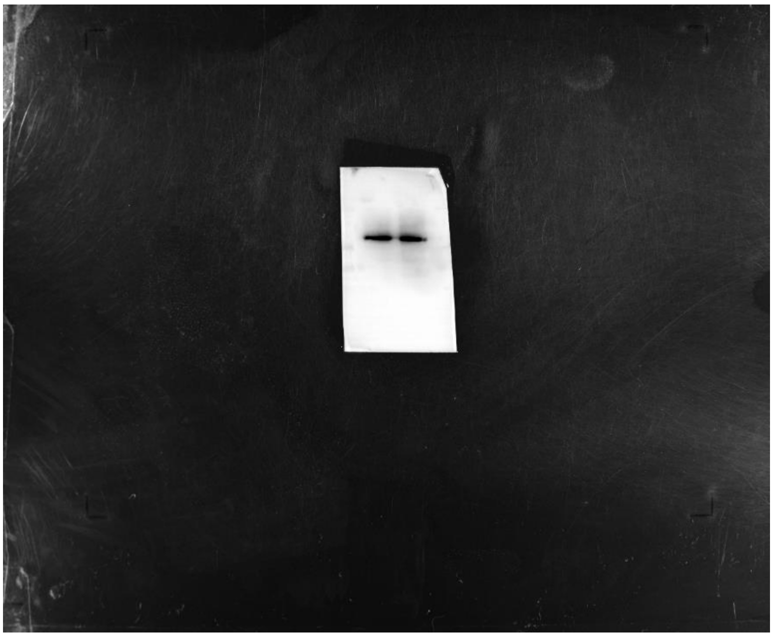

90kD

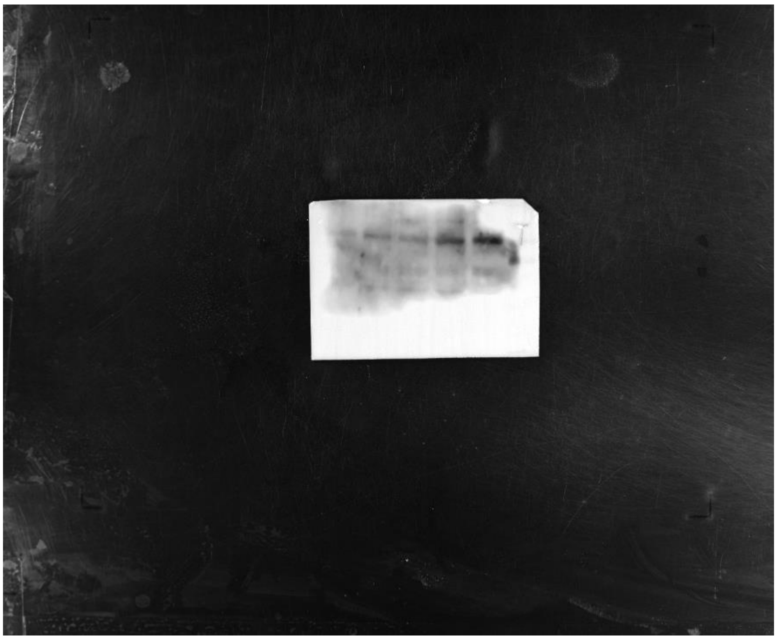

90kD

3.GPX4

Reference:

Liang D, Feng Y, Zandkarimi F, Wang H, Zhang Z, Kim J, Cai Y, Gu W, Stockwell BR, Jiang X. Ferroptosis surveillance independent of GPX4 and differentially regulated by sex hormones. Cell. 2023 Jun 22;186(13):2748-2764.e22. doi: 10.1016/j.cell.2023.05.003. Epub 2023 Jun 1. PMID: 37267948; PMCID: PMC10330611.

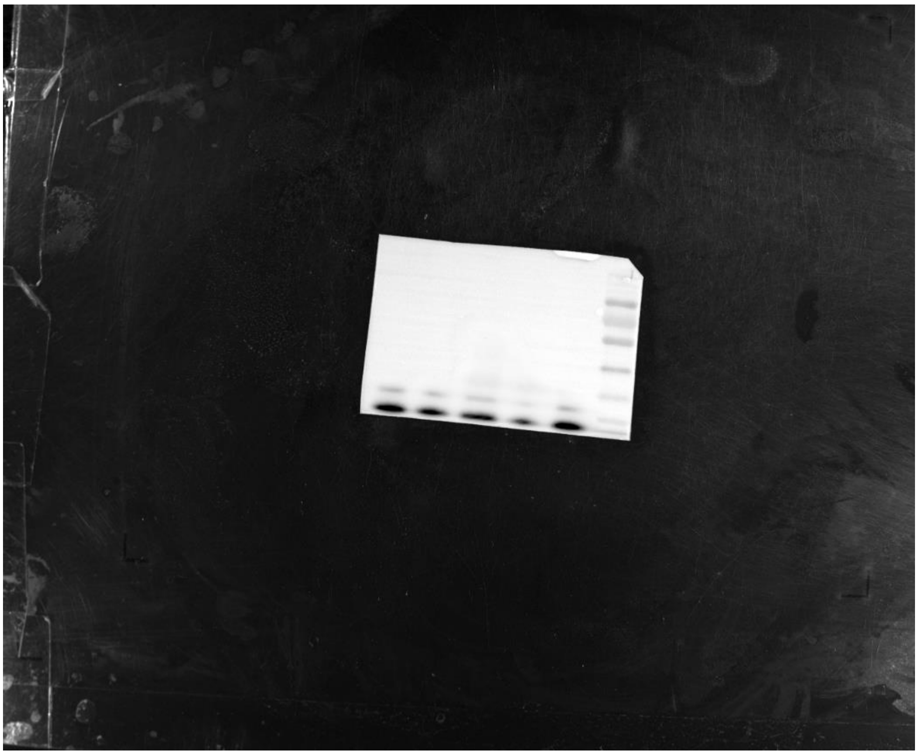

26kD

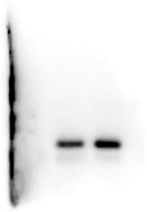

26kD

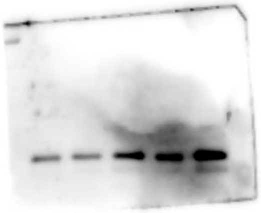

26kD

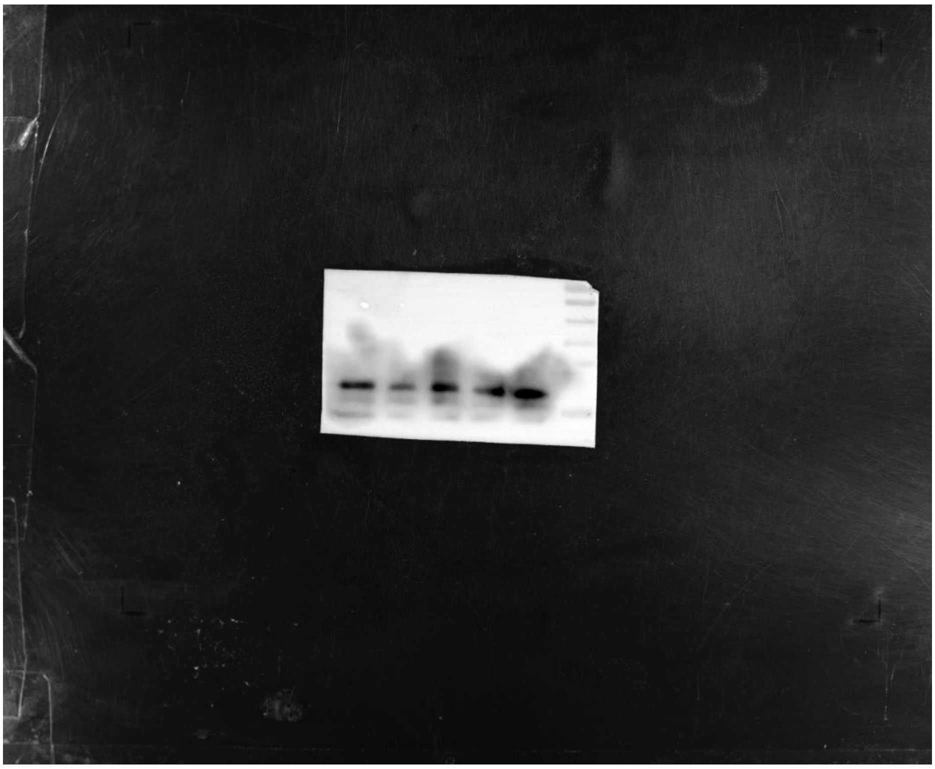

26kD

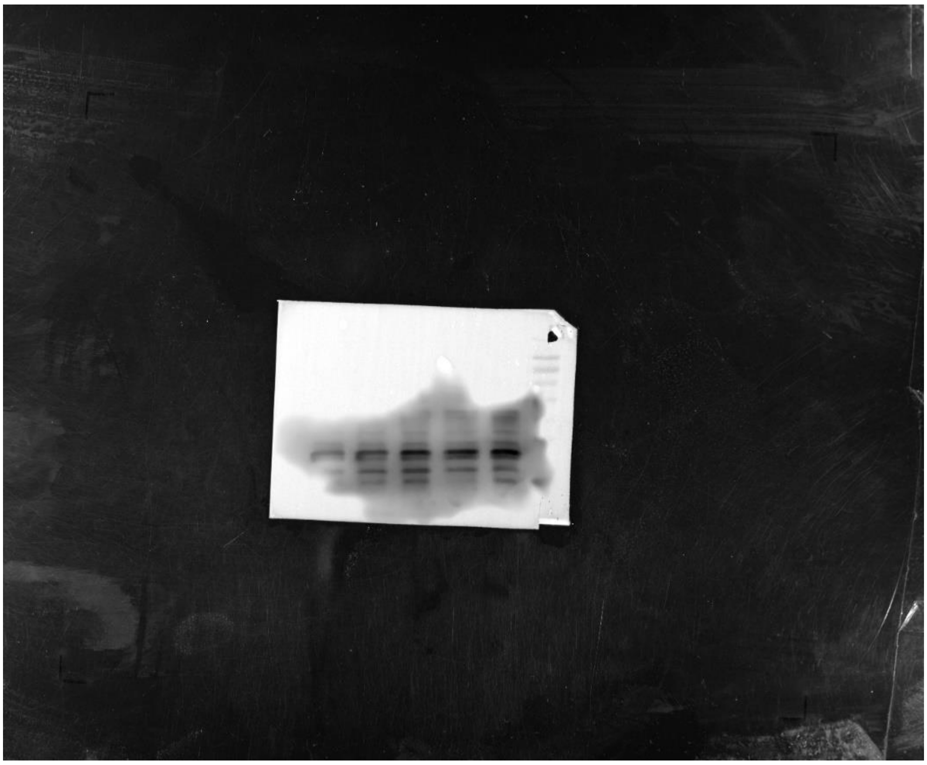

26kD

4.FTH1

Reference:

Kong N, Chen X, Feng J, Duan T, Liu S, Sun X, Chen P, Pan T, Yan L, Jin T, Xiang Y, Gao Q, Wen C, Ma W, Liu W, Zhang M, Yang Z, Wang W, Zhang R, Chen B, Xie T, Sui X, Tao W. Baicalin induces ferroptosis in bladder cancer cells by downregulating FTH1. Acta Pharm Sin B. 2021 Dec;11(12):4045-4054. doi: 10.1016/j.apsb.2021.03.036. Epub 2021 Mar 27. PMID: 35024325; PMCID: PMC8727776.

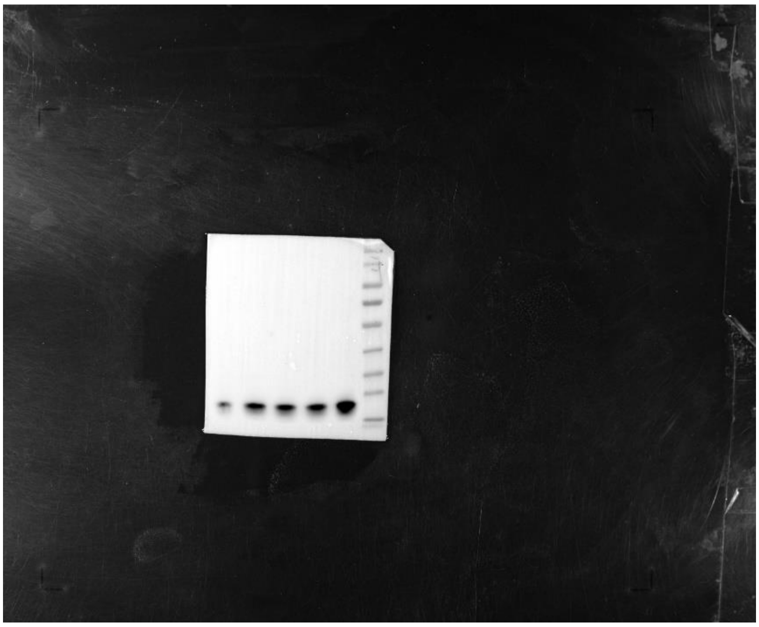

21kD

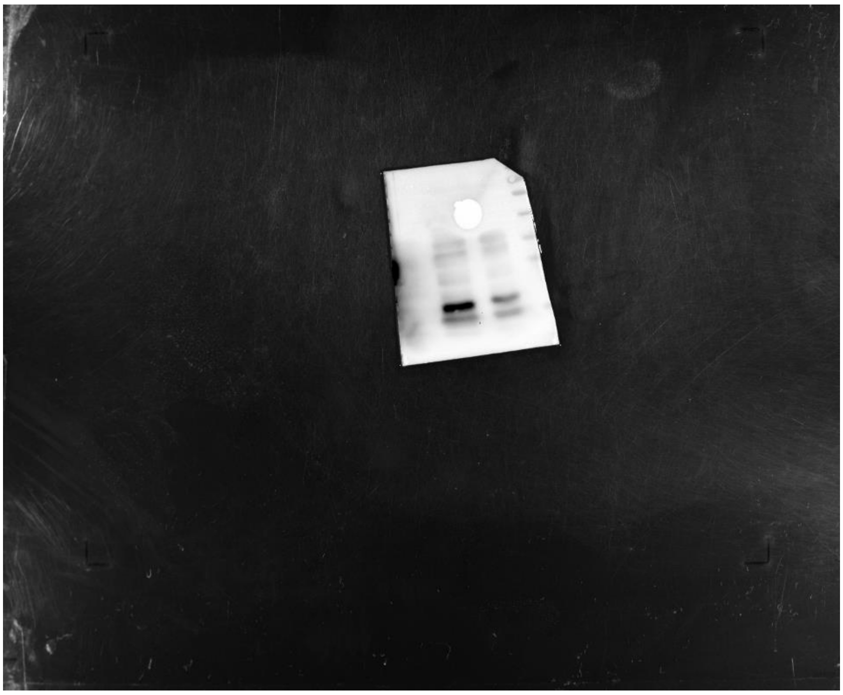

21kD

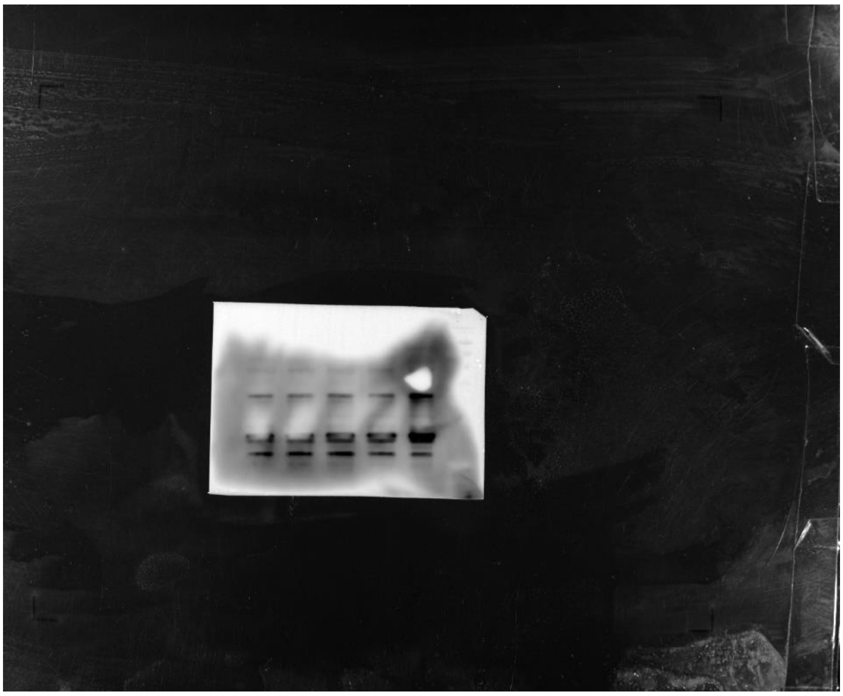

21kD

p53

Reference:

Xie Y, Hou W, Song X, Yu Y, Huang J, Sun X, Kang R, Tang D. Ferroptosis: process and function. Cell Death Differ. 2016 Mar;23(3):369-79. doi: 10.1038/cdd.2015.158. Epub 2016 Jan 22. PMID: 26794443; PMCID: PMC5072448.

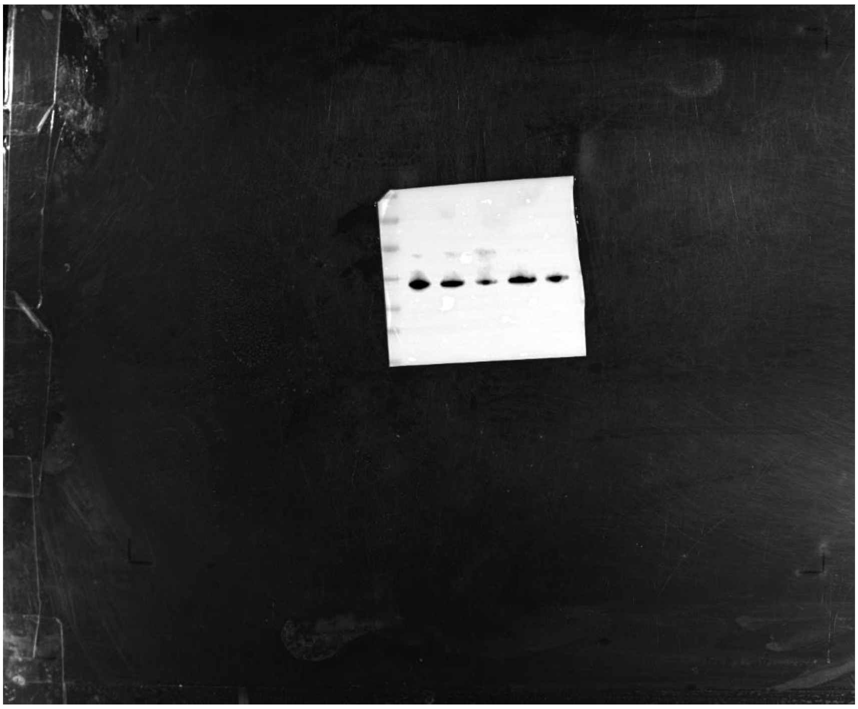

53KD

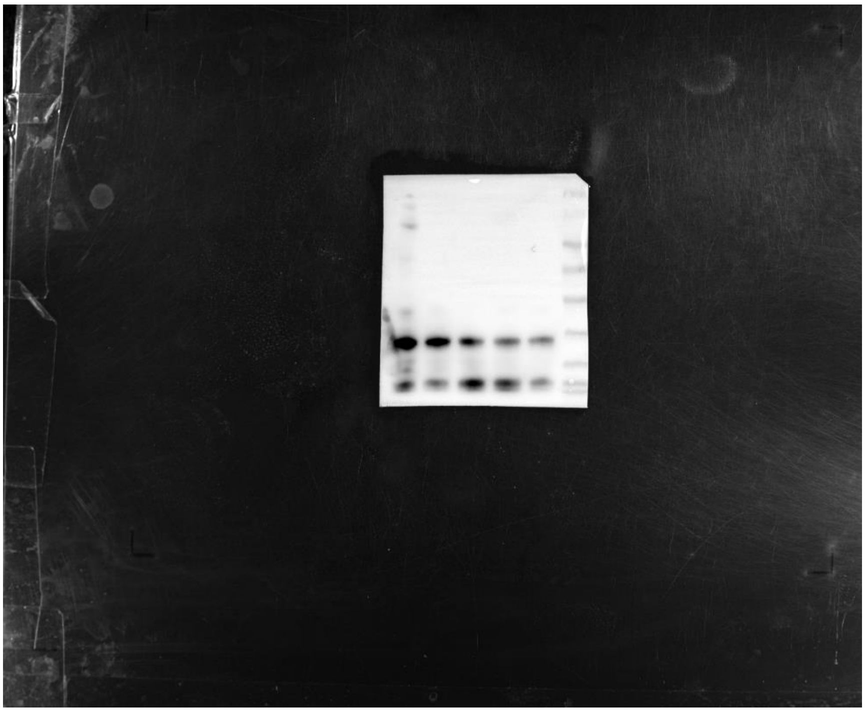

53KD

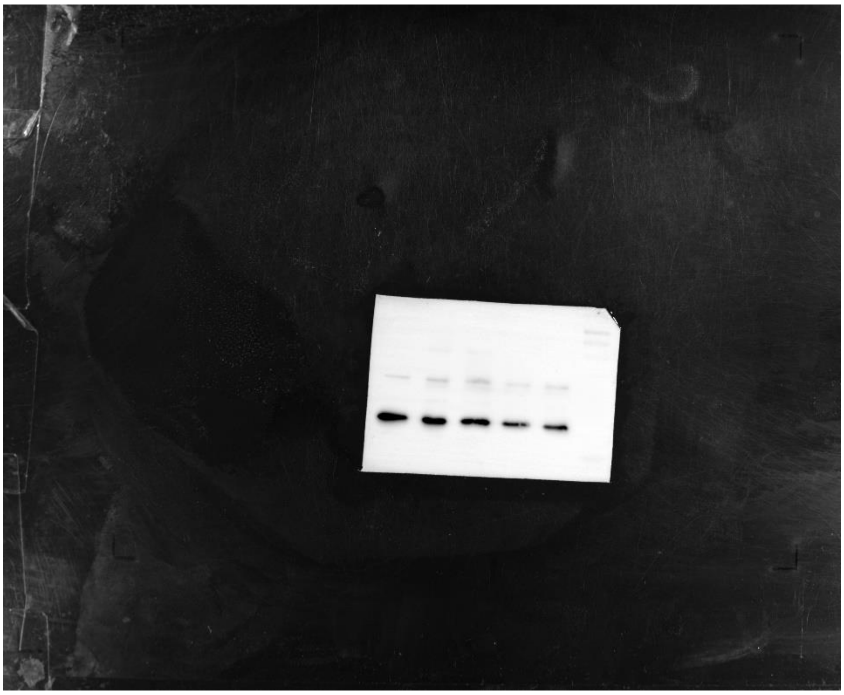

53KD

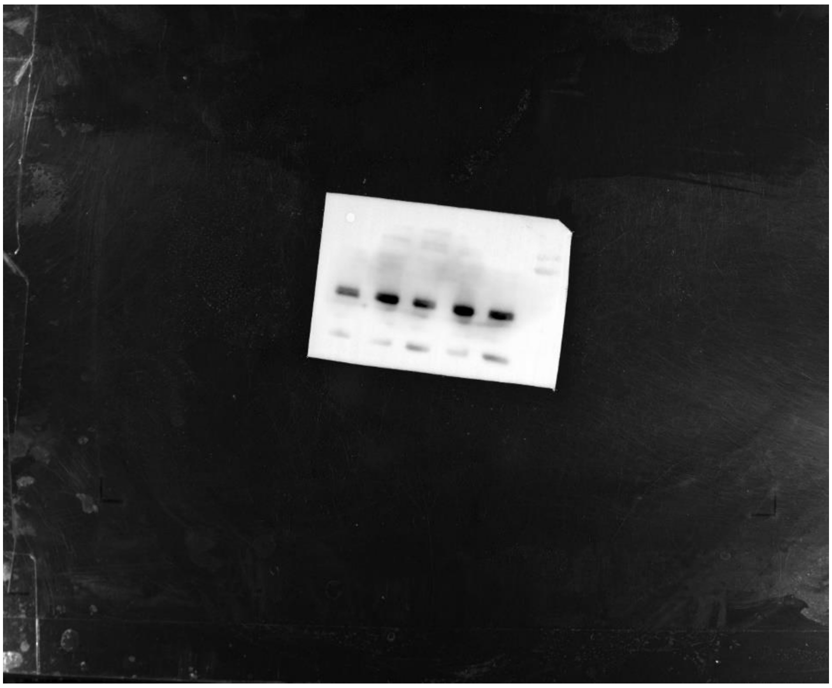

53KD

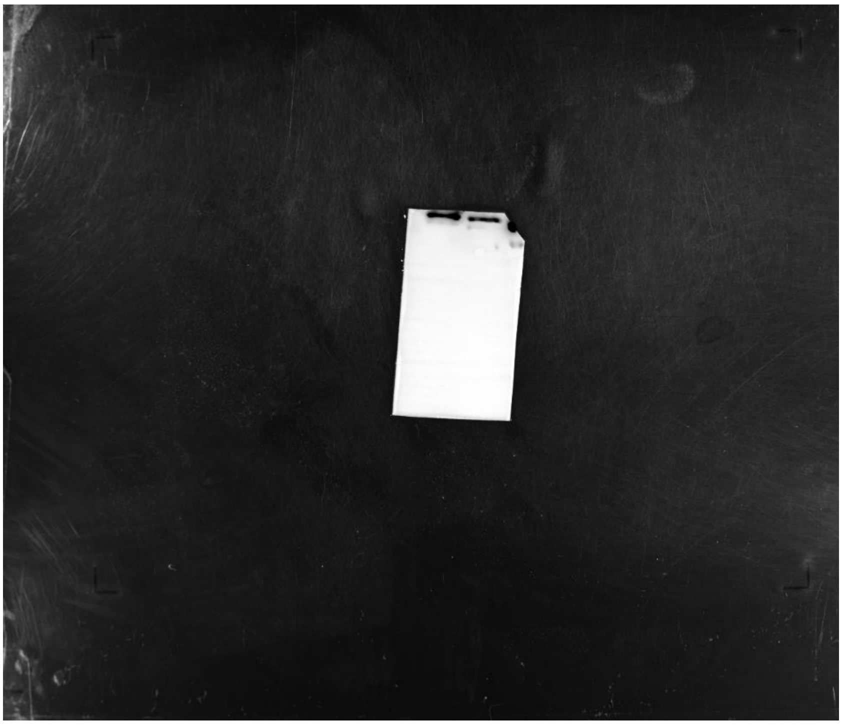

53KD

6.SLC7A11

Reference:

Koppula P, Zhuang L, Gan B. Cystine transporter SLC7A11/xCT in cancer: ferroptosis, nutrient dependency, and cancer therapy. Protein Cell. 2021 Aug;12(8):599-620. doi: 10.1007/s13238-020-00789-5. Epub 2020 Oct 1. PMID: 33000412; PMCID: PMC8310547.

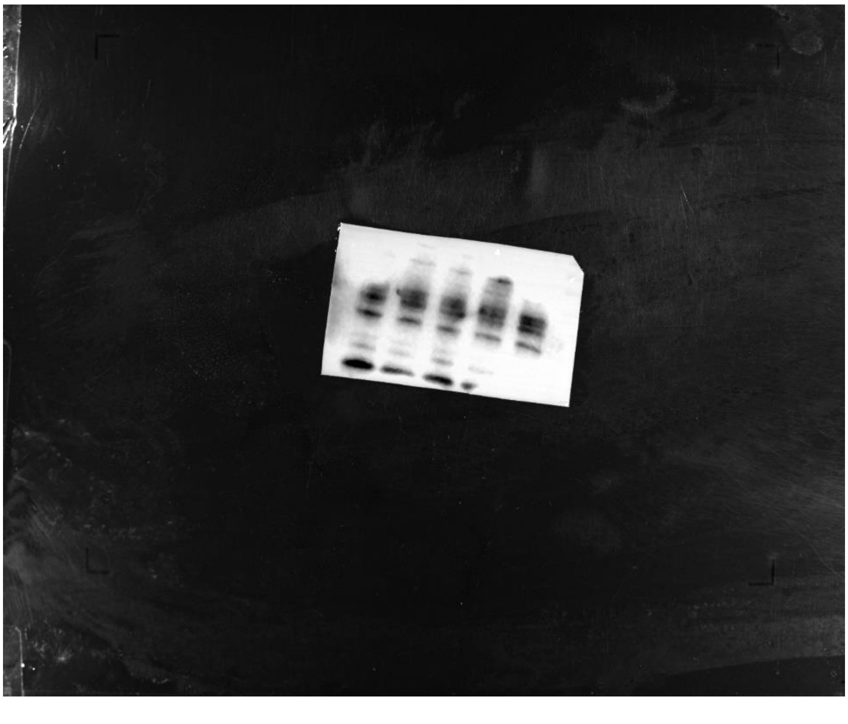

55KD

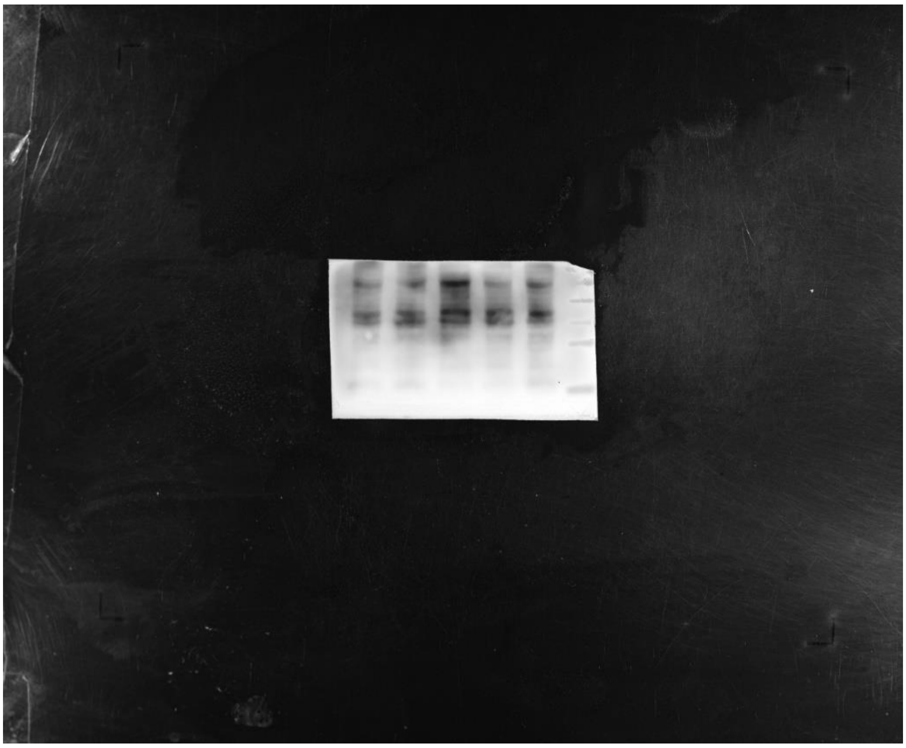

55KD

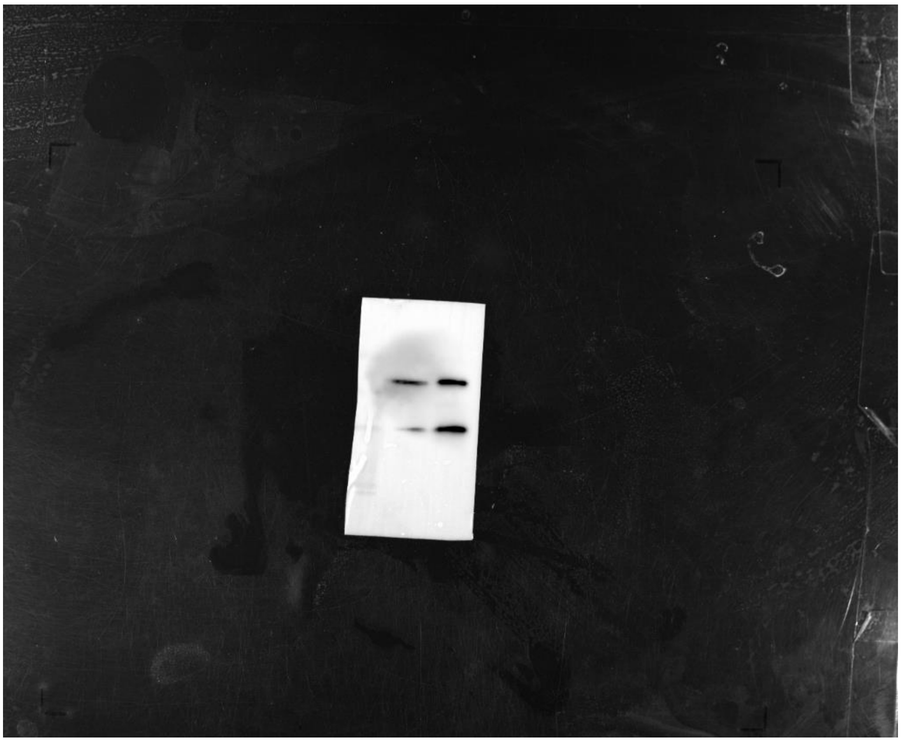

55KD

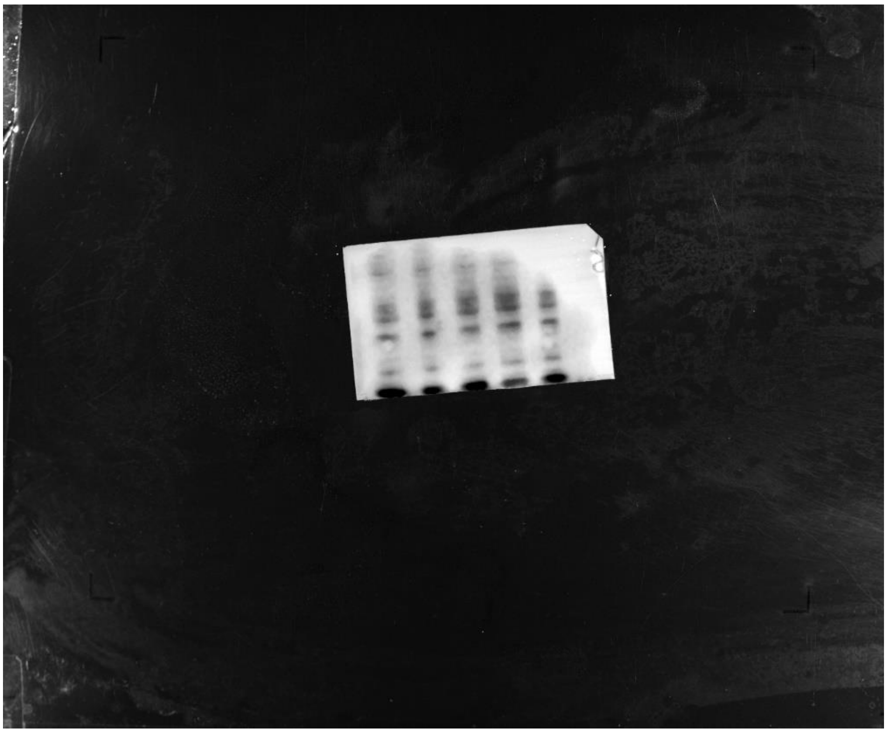

55KD

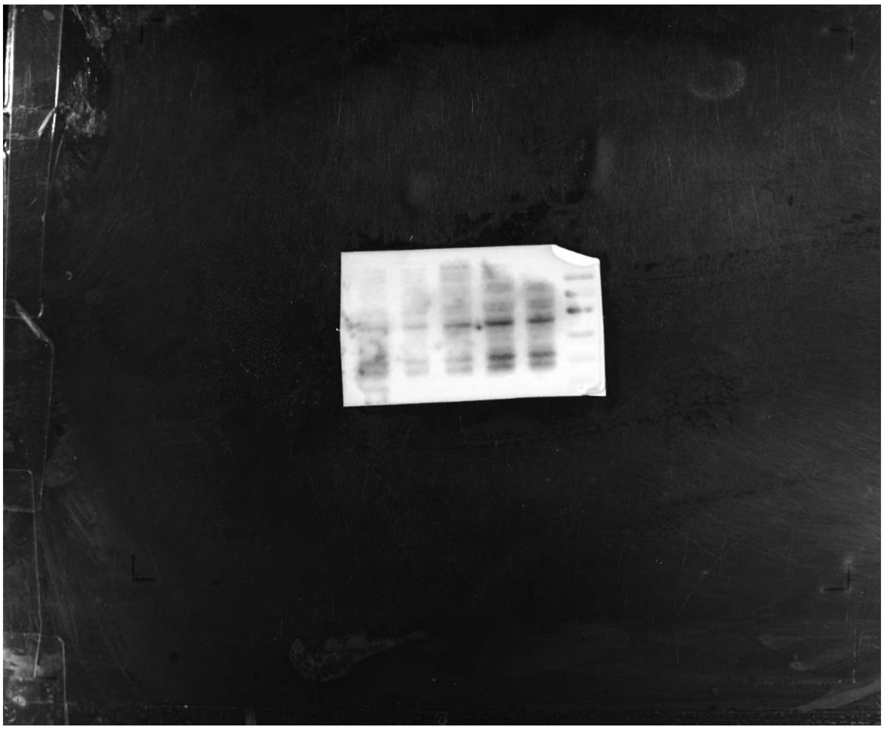

55KD

7.GAPDH

Shi M, Hou J, Liang W, Li Q, Shao S, Ci S, Shu C, Zhao X, Zhao S, Huang M, Wu C, Hu Z, He L, Guo Z, Pan F. GAPDH facilitates homologous recombination repair by stabilizing RAD51 in an HDAC1-dependent manner. EMBO Rep. 2023 Aug 3;24(8):e56437. doi: 10.15252/embr.202256437. Epub 2023 Jun 12. PMID: 37306047; PMCID: PMC10398663.

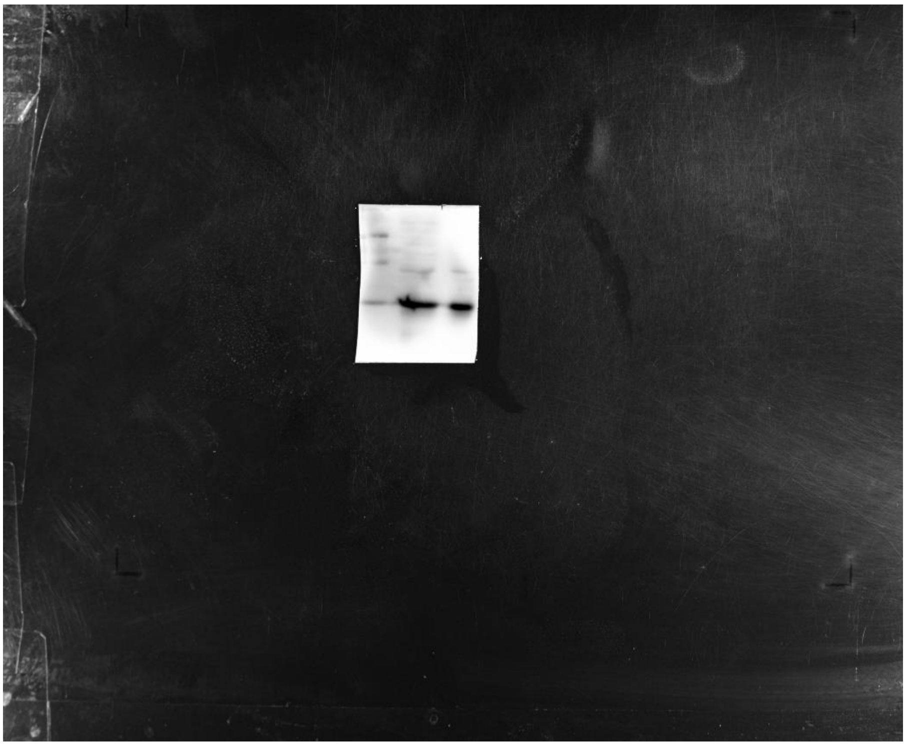

37KD

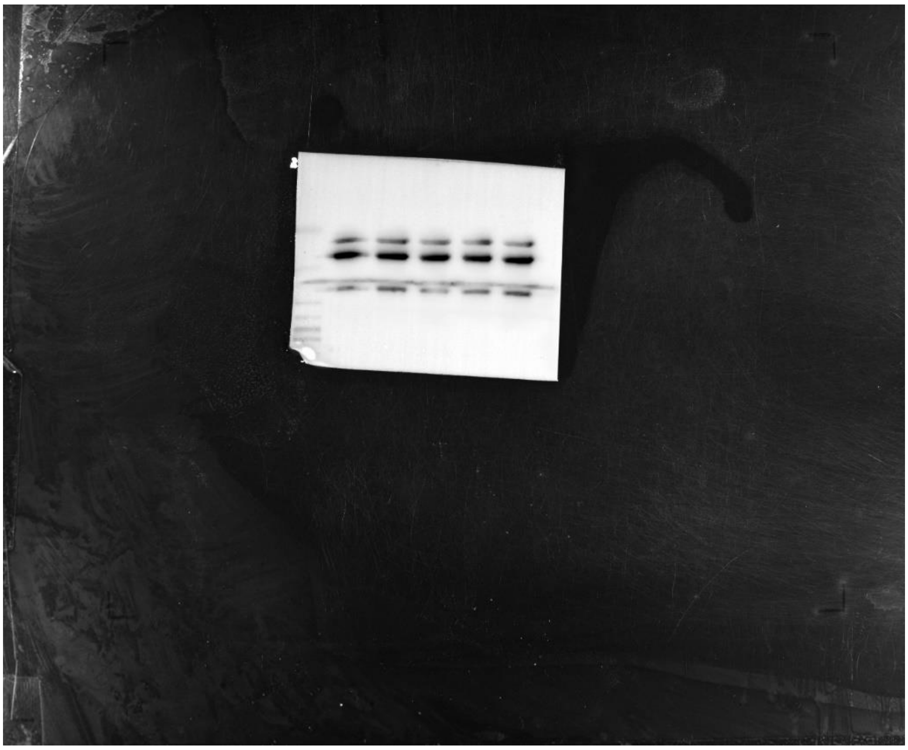

37KD

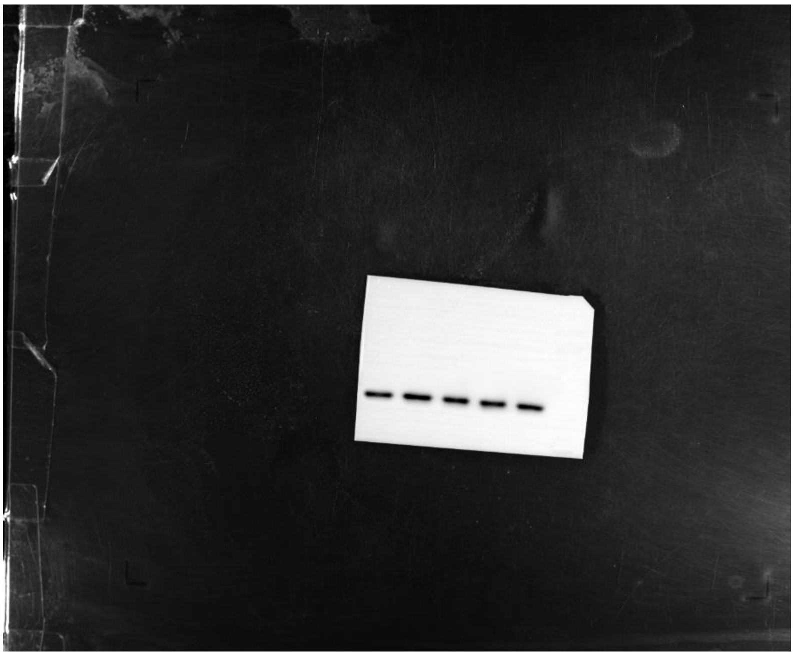

37KD

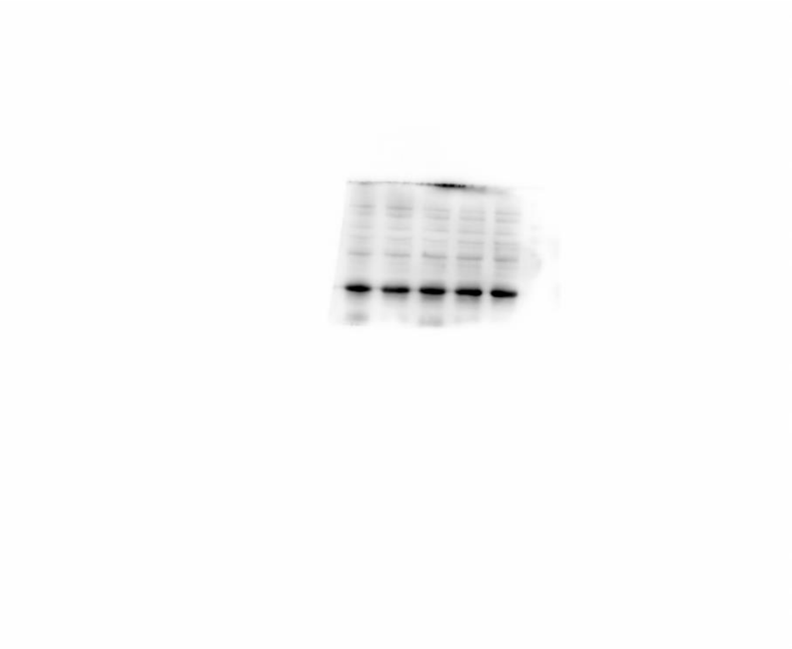

37KD

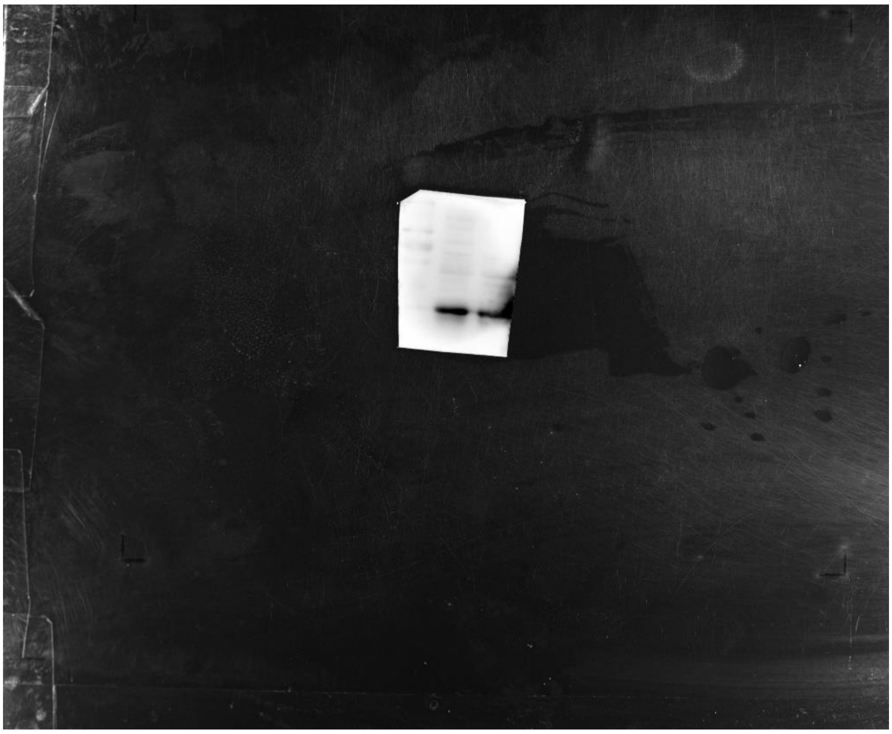

37KD

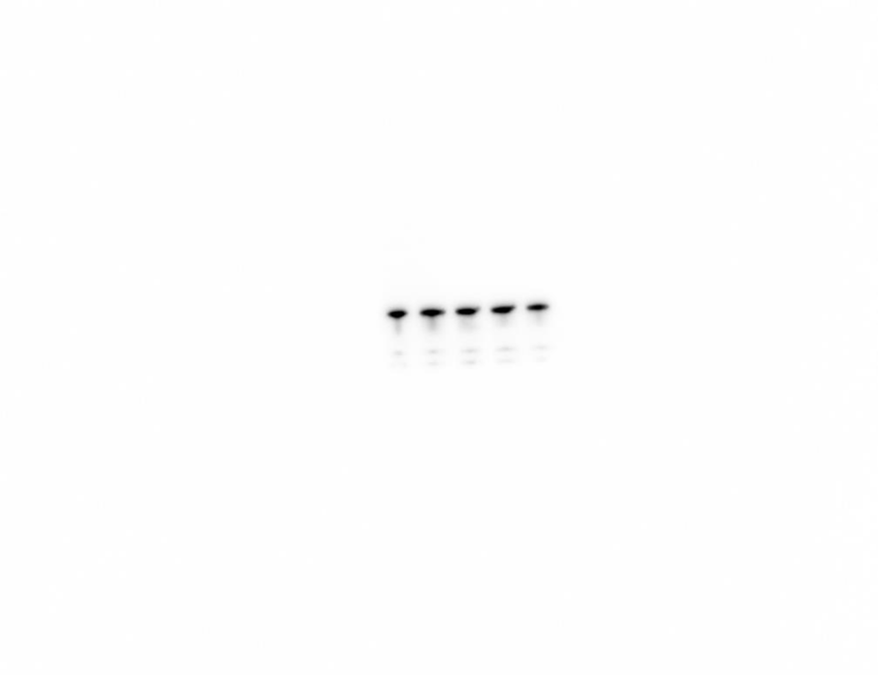

37KD

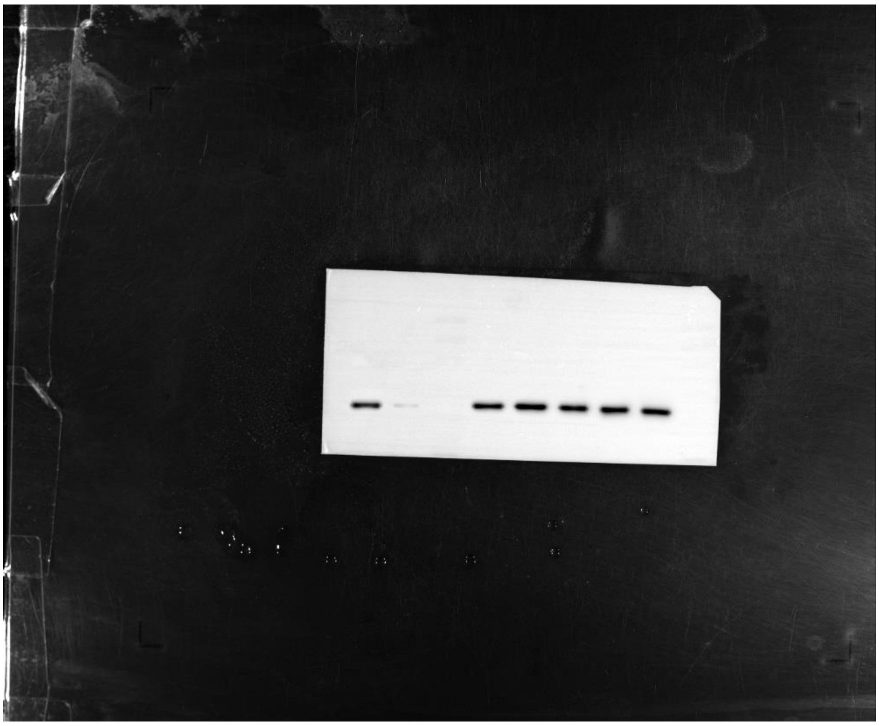

37KD

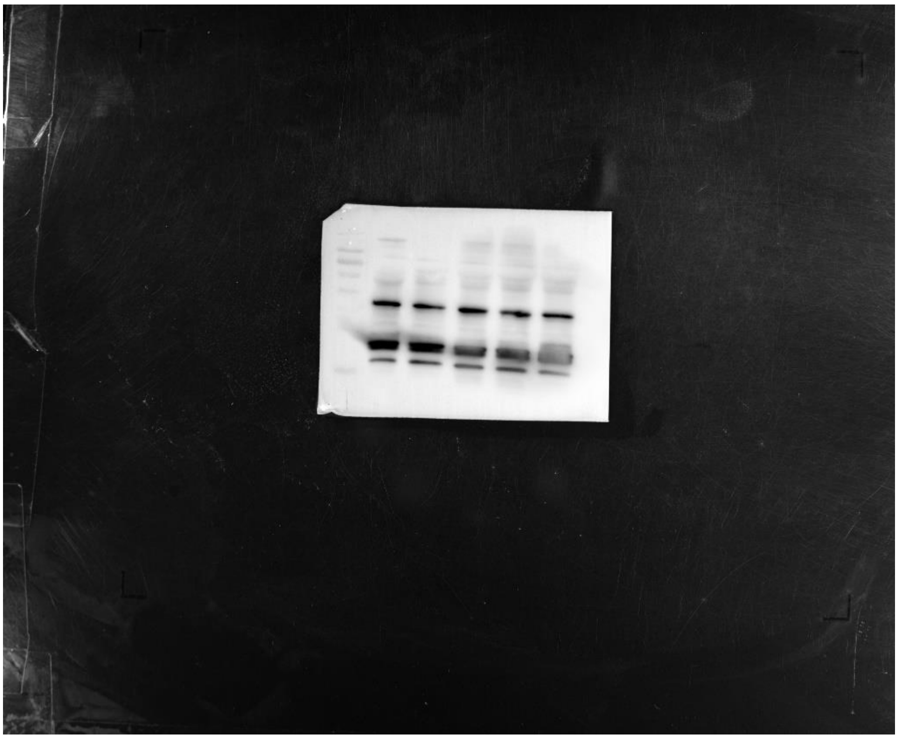

37KD
